# The Psychological Impact of Screen‐Detected Cancer: A Systematic Review

**DOI:** 10.1002/pon.70358

**Published:** 2026-01-23

**Authors:** Emma Lidington, Divyadharshini Ragupathy, Ninian Schmeising‐Barnes, Amanda Dibden, Jo Waller, Laura Marlow

**Affiliations:** ^1^ Centre for Cancer Screening, Prevention and Early Diagnosis, Wolfson Institute of Population Health Queen Mary University of London London UK; ^2^ School of Cancer and Pharmaceutical Sciences, Faculty of Life Sciences and Medicine King's College London London UK

**Keywords:** anxiety, cancer, early detection of cancer, mass screening, oncology, patient reported outcome measures, psychology

## Abstract

**Background:**

The benefits and harms of cancer screening must be balanced for all participant groups, including those who go on to have cancer diagnosed. The psychological impact of having cancer diagnosed through screening, rather than via another route, is currently unclear.

**Aims:**

We conducted a systematic review to describe the psychological impact of detecting cancer through screening (screen‐detected) compared to other routes (non‐screen‐detected).

**Methods:**

Eligible studies investigated the psychological impact of screen‐detected cancer. PubMed, Embase, and PsycINFO were searched. Two reviewers independently screened all titles, abstracts and full texts. We assessed quality using the Mixed Methods Appraisal Tool. Psychological outcome data were extracted for groups with screen‐detected and non‐screen‐detected cancers, calculating Cohen's *d* where relevant. Results were narratively synthesized.

**Results:**

We included 33 papers presenting quantitative results from 31 studies. All were considered medium to high quality. Studies measured psychological outcomes across six cancer screening programmes (breast, prostate, colorectal, lung, cervical and ovarian) using 31 different outcome measures. Receiving a screen‐detected cancer diagnosis seemed to be associated with a small or moderate short‐term increase in adverse psychological outcomes. In studies comparing outcomes by detection route, most found no difference (*n* = 16 studies), or that patients with screen‐detected cancers fared better than those with non‐screen‐detected cancers (*n* = 11 studies), but effect sizes were small.

**Conclusions:**

A screen‐detected cancer diagnosis can lead to short‐term adverse psychological outcomes; however, there is no strong evidence for a difference in psychological outcomes by detection route. Greater consistency of measures and timepoints would facilitate between‐study comparisons.

**PROSPERO registration:**

PROSPERO 2017 CRD42017075269.

## 1 Introduction

For most cancers, early‐stage diagnosis improves survival by increasing the chance of successful treatment [1, 2]. Screening provides an opportunity to detect cancer at an early stage by identifying individuals with cancer or a pre‐cancerous condition before symptoms occur [2, 3]. Evidence has shown that national screening programmes can reduce cancer‐specific mortality [4, 5]. Currently, the UK has population‐based screening programmes for bowel, breast and cervical cancers, and a targeted programme for those at elevated risk of lung cancer [3].

As screening is offered to ‘healthy’ individuals, rather than those seeking help for symptoms, the benefits must outweigh any potential harms [6]. Screening can result in false negative and false positive results and overdiagnosis (finding cancers that would never have caused harm). Overdiagnosis can lead to overtreatment with long‐term side‐effects and health sequelae, potentially reducing quality of life [7]. Furthermore, screening and diagnostic tests themselves can result in adverse events, causing short‐ or long‐term side‐effects [8].

Cancer screening can also bring psychological benefits and harms that must be considered. Potential positive outcomes include feelings of value and empowerment through the invitation and decision‐making process, reassurance among individuals with no cancer found or cancer found at an early stage, and potentially reduced decrements to quality of life by avoiding intensive treatment for late‐stage disease [9, 10, 11]. However, cancer screening, can also lead to increased anxiety, distress, and worry, particularly among people with indeterminant or false positive results [12]. Qualitative evidence also suggests that awareness of overdiagnosis and overtreatment can change an individual's sense of self, affect interactions with healthcare professionals and trigger feelings of regret [13].

For those diagnosed with cancer through screening, it is unclear how the psychological impact compares to cancer diagnosed through routine or urgent symptom investigation or incidental findings (non‐screen‐detected cancers). Some have hypothesised that screen‐detected diagnoses could cause additional harm, as screening participants without symptoms may be unaware of the possibility of disease [14]. This could result in shock, high levels of anxiety and lower trust in self‐awareness of symptoms and health [15]. A systematic review including people diagnosed with early‐stage prostate cancer suggested that a screen‐detected cancer diagnosis could lead to at least moderate psychological harm, although the studies included did not always report whether participants' cancers were screen‐detected [16].

Conversely, individuals diagnosed with screen‐detected cancer may be reassured by the increased curability of cancers detected early through screening, increasing hope for the future and resulting in relief and gratitude [15]. Moreover, diagnosing cancer through screening could also have positive psychological effects, due to the increased time and opportunity to control disease outcomes resulting in improved psychological wellbeing, especially in elderly patients [14].

To date, no reviews have specifically examined the psychological impact of screen‐detected cancer diagnoses. This review aims to fill this gap by examining the psychological impact of a cancer diagnosed through screening (*screen‐detected cancer*) and how this compares to cancer diagnosed through other routes (*non‐screen‐detected cancer*).

## 2 Methods

### 2.1 Design

This systematic review followed the Preferred Reporting Items for Systematic Review and Meta‐Analysis (PRISMA) guidelines and the Cochrane Handbook for Systematic Reviews of Interventions [17, 18]. The protocol was registered with PROSPERO, the International Prospective Register of Systematic Reviews, prior to starting the search (PROSPERO 2017 CRD42017075269).

### 2.2 Data Sources and Searches

PubMed/MEDLINE, PsycInfo and Embase were searched using OVID 16/12/2022 and updated on 30/07/2024. The search was restricted to English‐language papers and studies on humans. No restrictions were placed on publication date.

The search strategy was first created for PubMed and adapted for the other two databases. Medical Subject Headings (MeSH) terms and free‐text searches using truncations and Boolean operators were used. Briefly, search terms included: ‘cancer screening’, ‘positive’ and ‘diagnosis’, ‘prostate’, ‘lung’, ‘breast’, ‘colorectal’, ‘ovarian’ and ‘cervical’, ‘anxiety’, ‘depression’, ‘fear’, ‘worry’, and ‘psychological impact’. The full search strategies can be found in the Supporting Information.

### 2.3 Eligibility Criteria

Inclusion and exclusion criteria were agreed using the Population, Intervention, Comparison, Outcomes and Study (PICOS) design [18]. Studies were included if they collected and reported results for people with a screen‐detected breast, prostate, colorectal, lung, ovarian or cervical cancer. Breast, colorectal and cervical screening are widely recommended and targeted lung screening is increasingly being implemented. While not currently recommended, ovarian screening has been explored in many trials in which psychological outcomes are often measured. Similarly, prostate testing has been widely studied in large trials and though not widely recommended for screening, it is frequently offered to asymptomatic men in some countries. We decided to include these in the review because we would expect that a cancer diagnosis following ovarian or prostate testing when asymptomatic (even in a trial context) would be experienced in a similar way to that of a routine screening programme. This included studies that examined any type of screening tests for these cancers. No comparator group was required. Studies were included if they collected and reported any type of psychological outcome (e.g., anxiety, depression, distress, quality of life). Grey literature, literature reviews, narrative reviews, systematic reviews, meta‐analyses, conference abstracts, poster abstracts, editorial comments, letters, dissertations and book chapters were excluded.

Studies reporting qualitative and quantitative data were included in the initial search. Here, we report findings from the quantitative papers only.

### 2.4 Study Selection

Endnote X7 and Rayyan were used to manage the references. First, duplicates were removed manually. Then at least two researchers (DR, NSB or EL) independently screened each title and abstract. A third researcher resolved any discrepancies (DR, NSB, LM, JW or EL). Full‐text articles of papers meeting the inclusion criteria were also reviewed by at least two researchers (DR, NSB or EL). Where the full article could not be found or it was unclear from the article if it met the eligibility criteria, the authors were contacted. Backward citation searching was also conducted for papers included following the initial search check for additional papers.

### 2.5 Quality Assessment

The Mixed Methods Appraisal Tool (MMAT) was used to assess the quality of included articles [19]. Two reviewers rated each article (either EL, DR or NSB). Each reviewer independently categorised each paper into one of the five study designs following the user guide. Disagreements were resolved through discussion. Then the reviewers independently assessed each study against the relevant design‐specific quality criteria. Again, disagreements were resolved through discussion. Reporting an overall score is not necessarily recommended, but this has been provided to simplify presentation and interpretation according to the subsequent user guide guidance. Each paper was scored from 0% to 100% based on the proportion of criteria met. A score of 80%–100% was considered high, 40%–60% medium and 0%–20% low quality. Results are reported in Table 2, with a detailed breakdown in the supporting information.

### 2.6 Data Extraction

Data were extracted using a structured data collection form developed by DR, NSB, JW and LM in Google Sheets. The form was pilot tested with two studies and refined. One author (DR or EL) extracted the data for each study. The specific data extracted can be found in the Supporting Information. Where data were only presented in figures (e.g., confidence intervals presented as error bars), the data were estimated using an online digitizer app (PlotDigitizer, 3.1.6, 2025, https://plotdigitizer.com).

### 2.7 Data Synthesis

Due to the heterogeneity of outcomes, measures, time points and statistics presented, meta‐analysis was not possible. Instead, extracted data from each study are presented in a table and described in a narrative summary. We broadly categorised the concepts measured in each study to structure the presentation of results. When a study measured stress, worry or combined anxiety and depression, we categorised this as ‘psychological distress’. Where sufficient studies measured the same psychological construct with the same measure, results are presented graphically, showing time‐points.

Where papers reported psychological outcomes by cancer detection route (screen‐detected vs. non‐screen‐detected) we have described the differences. Cohen's *d* effect sizes were calculated to help interpret the size of differences found across studies using an online calculator (https://www.campbellcollaboration.org/calculator/). This was calculated using the sample size and frequency, percent or means and standard deviations. If not available, confidence intervals or standard errors were used to derive standard deviations following formulae reported by Cochrane [18]. Formulae provided by Chinn were used to convert odds ratios into Cohen's *d* effect sizes [52].

## 3 Results

**FIGURE 1:**
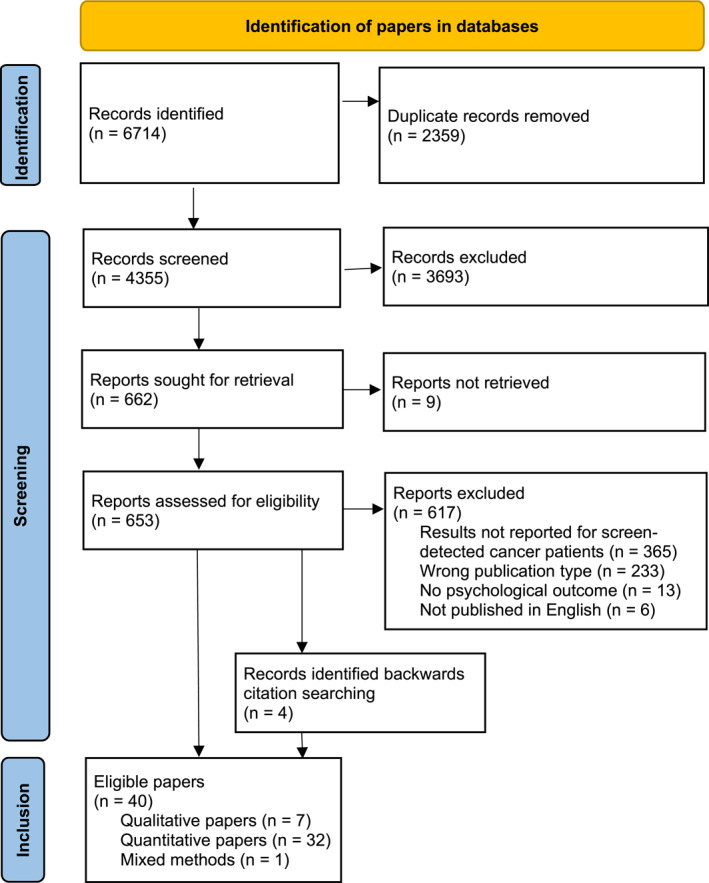
PRISMA diagram.

Initially, 6714 papers were identified through database searching (Figure 1). After duplicates were removed, 4355 titles and abstracts were screened. Of these, 653 papers were subject to full‐text review. Backward citation searching identified four further papers. In total, 33 papers presenting quantitative results from 31 studies were included. There were two instances where two separate papers reported the same study. Seven papers presenting qualitative results will be reported in a separate publication.

### 3.1 Study Characteristics

Summary characteristics for included studies can be found in Table 1. Twenty‐one papers presented longitudinal data and twelve cross‐sectional. Of the longitudinal studies, 19 were prospective and 2 were retrospective. Papers presented data from 10 countries including the United Kingdom (*n* = 9), Sweden (*n* = 4), the Netherlands (*n* = 4), the United States (*n* = 4), Spain (*n* = 3), Norway (*n* = 3), Canada (*n* = 1), Finland (*n* = 1) and Germany (*n* = 1).

**TABLE 1:** Summary characteristics of included studies.

First author (year);^a^ country	Type of cancer (screening test)	Psychological outcome (measure(s) used)	Study design	Data collection time‐points [2]	Non‐screen‐detected comparator group?	N Total	N screen detected cancers	N non‐screen‐detected cancers^b^	Quality appraisal
Barrett et al. (2014); England, Wales and Northern Ireland [29]	Ovarian cancer, serum CA125, transvaginal scan (TVS) or TVS ultrasound screening (USS)	Anxiety (state trait anxiety inventory, (STAI)), psychological morbidity (general health questionnaire, (GHQ‐12))	Longitudinal	Baseline prior to randomization; 6 weeks post‐surgery; 6 months post‐surgery	No	23374	*Not reported*	—	80% (high)
Booth et al. (2014); Finland [38]	Prostate, PSA testing	Quality of life (15D, EuroQual five dimension (EQ‐5D), short form six dimension (SF‐6D))	Longitudinal	After diagnosis; 1998 1999; 2003; 2011	Yes	5253	2883	2370	80% (high)
Braun et al. (2020); Germany [40]	Breast, mammography	Quality of life European Organisation for Research and Treatment of Cancer (EORTC) Quality of Life Questionnairen (QOL)‐C30 and QLQ‐BR23).	Cross‐sectional	Post‐diagnosis	Yes	735	346	389	100% (high)
Burgess et al. (2002); England [44]	Breast, mammography	Psychological morbidity (structured clinical interview to elicit psychiatric symptoms) including depression and anxiety	Longitudinal	5‐month post‐ diagnosis; 18‐month post‐diagnosis	Yes	132	52	80	80% (high)
Dang et al. (2019); The Netherlands [41]	Bowel, FIT test	Health status (EQ‐5d‐5L), quality of life (EORTC‐QLQ‐C30), fear of cancer recurrence (Cancer Worry Scale (CWS))	Cross‐sectional	0–42 months after treatment	No	110	110	—	80% (high)
Drummond et al. (2016); Ireland [34]	Prostate, PSA testing	Depression, anxiety, distress (Depression Anxiety Stress Scale‐21)	Cross sectional	2‐ To 8‐year post‐diagnosis	Yes	3348	1978	1331	80% (high)
Ekeberg et al. (2001); Norway [20]	Breast, mammography	Depression, anxiety (Hospital Anxiety and Depression Scale (HADS))	Longitudinal	day after recall mammography; 4‐week post diagnosis	No	213^b^	25	Not reported	60% (medium)
Ellman et al. (1989); England [42]	Breast, mammography	Psychological morbidity (GHQ‐28)	Longitudinal	Before screening; mammography; 3‐month post‐screening	Yes	733	18	20	80% (high)
Ellman et al. (1995); United Kingdom [21]	Breast, mammogram	Anxiety and depression (HADS)	Cross‐sectional	At mammogram follow‐up clinic or by post	Yes	915	205	126	80% (high)
Field et al. (2016); United Kingdom [22]	Lung, low dose CT	Lung cancer‐specific distress (Cancer Worry Scale—Revised (CWS‐R)), anxiety and depression (HADS), decision satisfaction (satisfaction and decision scale),	Longitudinal	Prior to screening; 2 weeks after CT result or control notification; 10–27 months after initial recruitment	No	4027	23	—	80% (high)
Fortin et al. (2023); Canada [49]	Breast, not indicated	Distress (distress thermometer)	Cross‐sectional	Post diagnosis/treatment	No	18	7	11	40% (medium)
Gareen et al. (2014); United States [30]	Lung, low dose computed tomography and chest x‐ray	Quality of life (SF‐36 MCS), anxiety (STAI)	Longitudinal	At screening (SF‐MCS only); 1‐month after screening; 6‐month after screening	No	1239	63	—	80% (high)
Gibbons et al. (2016); Ireland [24]	Breast, mammography	Depression, anxiety, (HADS) distress (cancer‐related distress)	Cross‐sectional	At diagnosis	No	94	94		60% (medium)
Gibbons et al. (2017); Ireland [23]	Breast, mammography	Depression, anxiety, (HADS) distress (Perceived Stress Scale, Impact of Events Scale)	Longitudinal	At diagnosis; 12 months post diagnosis	Yes	221	92	129	40% (medium)
Gustafsson et al. (1995); Sweden [48]	Prostate, PSA testing	Stress (cortisol), sleep (Sleep Disturbance Index)	Longitudinal	At screening; 2‐week after screening; 4‐week after screening; 16‐week after screening	No	1782	33	—	40% (medium)
Haddad et al. (1994); England [45]	Breast, mammography	Psychological morbidity ‐ (psychiatric assessment schedule): Major depression and/or generalised anxiety, adjustment disorder	Longitudinal	2‐month post‐diagnosis; fourteen months post‐diagnosis	Yes	295	82	213	100% (high)
Lampic et al. (2002); Sweden [26]	Breast, mammogram	Anxiety and depression (HADS), life values (The Life Value Questionnaire)	Longitudinal	At time of recall; A few days after recall visit; 3 months post recall; 1 year post recall; 2 years post recall	No	517	45	—	80% (high)
Lampic et al. (2001); Sweden [25]	Breast, mammogram	Anxiety and depression (HADS),	Longitudinal	At time of recall; A few days after recall visit; 3 months post recall; 12 months post recall	No	509	44	—	60% (medium)
Lu et al. (2019); Sweden [46]	Cervical, not indicated	Stress related disorders, stressful life events	Cohort	In the year prior to cervical cancer diagnosis	Yes	4245	1241	3004	100% (high)
McSweeney et al. (2017); Ireland [27]	Breast, mammography	Global distress/depression, anxiety (HADS)	Cross‐sectional	Post diagnosis	Yes	93	48	45	80% (high)
Menon et al. (2023); United Kingdom [43]	Ovarian, ultrasound/Multimodal screening (CA125 testing + transvaginal scan)	Anxiety (STAI), psychological morbidity (GHQ‐12), health status (Functional Assessment Of Cancer Therapy—Ovarian (FACT‐O)), quality of life (EQ‐5D)	Randomised control trial	Baseline prior to randomisation; 6 weeks post‐surgery (if having surgery); 6 months post‐surgery (if having surgery‐ then left the study)	No	202,638 (randomised to screening or control), screened = 101,279	1042	1016 *[no psychological outcome data collected for this group]*	80% (high)
Miles et al. (2015); Scotland [36]	Colorectal, faecal occult blood test	Depression (Centre for Epidemiologic studies depression) scale (CES‐D)), quality of life (Functional Assessment of cancer therapy—Colorectal (FACT‐C))	Cross‐sectional	6–8 years post‐diagnosis	Yes	296	106	99	80% (high)
Orbell et al. (2008); United Kingdom [53]	Colorectal	Anxiety (STAI), coping (Ways Of Coping—revised), Illness Perceptions Questionnaire ‐ revised (adapted IPQ‐R)	Longitudinal	Post‐treatment	No	697	196	—	60% (medium)
Politi et al. (2020); Spain [47]	Breast, mammography	Psychological events: Fatigue, anxiety, depression (hospital and HC3 chart review)	Cross‐sectional	3 months post‐surgery until disease free	Yes	211	134	77	80% (high)
Roth et al. (2009); United States [37]	Breast, mammography	Depression (CES‐D)	Longitudinal	At diagnosis; 12‐month post‐diagnosis	Yes	86	11	75	80% (high)
Solbjør et al. (2018); Norway [51]	Breast, mammography	Anxiety, dejection, negative impact on behaviour, negative impact on sleep, sexuality, calm/relaxed, social network, existential values Psychological Consequences Questionnaire‐ Breast Cancer (PCQ‐BC)	Longitudinal	When receiving screening result; 1 month after screening; 6‐month after screening	No	388	28	—	80% (high)
Schou Bredal et al. (2013); Norway [28]	Breast, mammography	Anxiety and depression (HADS)	Longitudinal	On the day of recall; 4 weeks after results	No	640	79	—	80% (high)
Van den Bergh et al. (2012); Netherlands [31]	Prostate, not indicated	Quality of life (SF‐12 (Mental Component Summary)), depression (CES‐D), anxiety (STAI)	Longitudinal	6 months post diagnosis/treatment; 18 months post diagnosis/treatment	No	266	266	—	80% (high)
Van der Steeg et al. (2011); Netherlands [32]	Breast, mammography	Quality of life (World Health Organisation Quality of Life −100 item measure (WHOQOL 100)), personality (NEO‐FFI), anxiety (STAI)	Longitudinal	Prior to diagnosis; at diagnosis; 1‐month post‐diagnosis; 3‐month post‐diagnosis; 6‐month post‐diagnosis	No	385	152	—	80% (high)
Varela‐Moreno et al. (2022); Spain [35]	Colorectal, not indicated	Depression (HADS, adapted)	Cross‐sectional	5‐year post follow up assessment	Yes	2602	429	1851	100% (high)
Venderbos et al. (2017); Netherlands [33]	Prostate, PSA	Prostate specific health (Expanded Prostate Cancer Index Composite (EPIC)), generic health: QoL (SF‐12, EQ‐VAS) (analogue scale), anxiety (STAI).	Cross‐sectional	6–8.6 years post diagnosis	No	618	69	Not reported	80% (high)
Vermeer et al. (2020); United States [39]	Colorectal, faecal occult blood test	Worry (CWS), quality of life (HRQoL) screening related distress (PCQ), decision regret (Decision Regret Scale, DRS)	Longitudinal	Before colonoscopy; post‐result notification; 6‐month post‐result	Yes	4842	69	205	60% (medium)
Yang et al. (2023); United States [50]	Breast, mammogram	Distress (Common Cancer Concerns Questionnaire)	Cross‐sectional	Initial visit after diagnosis	Yes	745	401	344	60% (medium)

^a^ Some articles reported the results from the same trials/studies at different time points/with additional information: Menon et al., 2023 [43] and Barrett et al., 2014 [29]; Lampic et al., 2001 [25] and Lampic et al., 2002 [26].

^b^ including symptomatically detected and interval cancers.

**TABLE 2:** Results of included studies for screen‐detected and non‐screen‐detected cancers.

Author (year), cancer type	Outcome measure	Time points (*n*)	Results for baseline (where applicable) and screen detected cancer group	Results for non‐screen detected group(s)	Difference by mode of detection, [effect size (Cohen's D)]
ANXIETY					
Hospital Anxiety and Depression Scale—Anxiety Component (HADS‐A)					
Ekeberg (2001)^+^, Breast [20]	HADS‐A: Seven items scored 0–3, range 0–21. ≥ 11 indicates anxiety case.	1. At recall (*n* = 25) 2. 4 weeks post‐diagnosis (*n* = 25)	1. *M* = 8.83 (SD = 4.6) 2. *M* = 7.76 (SD = 4.9) Total SnD cancer group: 1. % > 11 = 28% 2. % > 11 = 24%	—	—
Ellman (1995), Breast [21]	HADS‐A: Scoring same as Ekeberg. No cut‐off.	Completed in follow‐up clinic by long‐term survivors (any time from diagnosis) (SnD: *n* = 205; non‐SnD: *n* = 126)	Not presented for SnD alone	Not presented for non‐SnD alone	No significant difference (data not shown) [insufficient data to calculate]
Field (2016), lung [22]	HADS‐A: Scoring same as Ekeberg.	1. Baseline before CT scan (people later invited to screening *n* = 2018) 2. Two weeks after initial scan (positive referrals, *n* = 48) 3. 10–27 months later (true positives, *n* = 23)	1. *M* = 3.72 (CI not presented) 2. *M* = 5.49 (CI = 4.48–6.67) 3. *M* = 2.94 (CI = 2.08–4.03)	—	—
Gibbons (2017), Breast [23]	HADS‐A: Scoring and cut‐off same as Ekeberg.	1. At diagnosis 2. 12 months post‐diagnosis (At both time points—SnD: *n* = 92; non‐SnD: *n* = 129)	1. *M* = 7.70 (SD = 4.08) *N* > 11 = 28 (30.4%) 2. *M* = 6.40 (SD = 4.25) *N* > 11 = 8 (8.7%)	1. *M* = 8.97 (SD = 5.07) *N* > 11 = 46 (35.6%) 2. *M* = 5.76 (SD = 3.68) *N* > 11 = 10 (7.8%)	No significant difference 1. *χ*2 = 2.33, df = 2, *p* = 0.313 2. *χ*2 = 1.56, df = 2, *p* = 0.458 [SnD versus non‐SnD: 1. −0.27; 2. 0.16]
Gibbons (2016), Breast [24]	HADS‐A: Scoring and cut‐off same as Ekeberg.	After dx/before tx (*n* = 94)	*M* = 7.97 (SD = 4.48) *N* > 11 = 28 (30.4%)	—	—
Lampic (2001)*, Breast [25]	HADS‐A: Scoring and cut‐off same as Ekeberg.	1. Prior to recall (*n* = 509) 2. A few days after recall (*n* = 34) 3. 3 months after recall (*n* = 34) 4. 1 year after recall (*n* = 31)	1. *M* = 7.4 (SD = 4.3) 2. *M* = 7.6 (SD = 5.5) 3. *M* = 6.1 (SD = 5.3) 4. *M* = 5.9 (SD = 5.3)	—	—
Lampic (2002)*, Breast [26]	HADS‐A: scoring same as Ekeberg. > 8 indicates clinical anxiety.	1. 3 months after recall (*n* = 34) 2. 1 year after recall (*n* = 31)	1.% > 8 = 26% 2. % > 8 = 32%	—	—
McSweeney (2017), Breast [27]	HADS‐A: scoring and cut‐off same as Ekeberg.	Not stated (long‐term survivors) (*n* = 48)	*N* > 11 = 21 (43.8%)	*N* > 11 = 23 (51.1%) (*n* = 45)	No significant difference (data not shown) SnD versus non‐SnD; −0.16
Schou Bredal (2013), Breast [28]	HADS‐A: scoring same as Ekeberg. > 11 indicates clinically relevant anxiety.	1. At recall (*n* = 80) 2. After results (*n* = 72)	1. *M* = 6.6 (SD = 3.9) *n* > 11 = 18.8% 2. *M* = 5.6 (SD = 3.9) *n* > 11 = 16.7%	—	—
State‐Trait Anxiety Inventory (STAI)					
Barrett (2014), Ovarian [29]	STAI‐20 (state scale): 20 items, treated as continuous variables. Total score 20–80.	1. Screening appointment 2. 6 weeks post‐surgery 3. 6 months post‐surgery (*n* = not reported)	Mean change from screening appointment: 2. +3.21 (CI 1.62,4.80) 3. −0.18 (CI −1.68, 1.32)	—	—
Gareen (2014)^+^, lung [30]	STAI‐20 (state scale): Total score 20–80. Median norm for 50–69 year old men is 34.51, women 32.20.	1. 1‐month post‐screening (*n* = 48) 2. 6‐month post‐screening *(n = 42)* *not all received diagnosis by time of survey	1. *M* = 40.5 (SD = 14.0) 2. *M* = 38.3 (SD = 12.9)	—	—
Van den Bergh (2012)^+^, Prostate [31]	STAI‐6 (score range 20–80 (maximum anxiety), threshold for high anxiety: > 44).	1. 6‐month post‐diagnosis (*n* = 266) 2. 18‐month post‐diagnosis (*n* = 215)	1. *M* = 33.9 2. *M* = 33.3	—	—
van der Steeg (2011), Breast [32]	STAI (version, scoring and interpretation not described).	1. Before diagnosis 2. At diagnosis 3. 1‐month post‐diagnosis 4. 3‐month post‐diagnosis 5. 6‐month post‐diagnosis 6. 12‐month post‐diagnosis (*n* = 152, not reported per timepoint)	1. *M* = 48.3 (SD = 13.9) 2. *M* = 48.1 (SD = 14.8) 3. *M* = 37.0 (SD = 11.1) 4. *M* = 36.6 (SD = 12.8) 5. *M* = 35.7 (SD = 12.7) 6. *M* = 35.4 (SD = 11.5)	—	—
Venderbos (2017), prostate [33]	STAI‐6: Scored 20–80; higher scores indicated more anxiety; ≥ 44 indicates high anxiety.	6–8 years post diagnosis (*n* = 70)	*M* = 33.5 (SD = 10.5) *N* ≥ 44 = 9 (13%)	—	—
Depression, Anxiety, and Stress Scale (DASS)—Anxiety Scale					
Drummond (2016), prostate [34]	DASS (anxiety scale): 7 items, 4‐point likert scale, 0–3. Total score—0–21. > 8 indicates anxiety.	2–18 years post‐diagnosis (SnD: *n* = 1978; clinically detected: *n* = 1331)	*N* ≥ 8 = 14% (CI = 12%–15.2%)	*N* ≥ 8 = 21% (CI = 19.3%–22.5%)	Screen‐detected significantly lower: *p* < 0.001 (*t*‐test) [SnD versus non‐SnD: −0.27]
Depression					
Hospital Anxiety and Depression Scale—Depression Component (HADS‐D)					
Ekeberg (2001)^+^, Breast [20]	HADS‐D: Seven items scored 0–3. Score calculated by simple addition, higher score indicates higher anxiety. ≥ 11 indicates ‘caseness’. Total score range: 0–21.	1. At recall (*n* = 25) 2. 4 weeks post‐diagnosis (*n* = 25)	1. *M* = 4.0 (SD = 3.4); *N* > 11 = 1 (3.8%) 2. *M* = 4.3 (SD = 4.4); *N* > 11 = 3 (12%)	1. *N* > 11 = 1 (3.8%) 2. *N* > 11 = 3 (12%)	[Insufficient data to calculate]
Ellman (1995), Breast [21]	HADS‐D: 7 items, scores calculated by simple addition, scores of 7/8 indicates borderline and 10/11 indicates definite depression. Total score range 0–21.	Not stated (long‐term survivors) (SnD: *n* = 205; non‐SND: *n* = 126)	Not presented for SnD alone	—	No significant difference (data not shown) [Insufficient data to calculate]
Field (2016), lung [22]	HADS‐D: scoring same as above. Score ≥ 11 moderate, ≥ 14 severe.	1. Baseline before CT scan (people later invited to screening *n* = 2018) 2. Two weeks after initial scan (positive referrals, *n* = 48) 3. 10–27 months later (true positives, *n* = 23)	1. *M* = 2.66 2. *M* = 3.05 (CI = 2.44–3.78) 3. *M* = 2.52 (CI = 1.79–3.44)	—	—
Gibbons (2017), breast [23]	HADS‐D: scoring same as above.	1. At diagnosis 2. 12 months post‐diagnosis (At both timepoints ‐ SnD: *n* = 92; non‐SnD: *n* = 129)	1. Mean = 3.38 (SD = 3.19) *N* > 11 = 6 (6.5%) 2. Mean = 3.36 (SD = 4.28) *N* > 11 = 3 (3.3%)	1. Mean = 4.37 (SD = 3.89) > 11 = 11 (8.5%) 2. Mean = 3.58; SD = 3.19; > 11 = 4 (3.1%)	No significant difference: 1. *χ*2 = 3.35, df = 2, *p* = 0.187 2. *χ*2 = 1.69, df = 2, *p* = 0.430 [SnD versus non‐SnD: 1. −0.27 2. −0.06]
Gibbons (2016), breast [24]	HADS‐D: scoring same as above.	After dx/before tx (*n* = 94)	Mean = 4.05 (SD = 3.72) > 11 = 6 (6.5%)		—
Lampic (2001)*, breast [25]	HADS‐D: scoring same as above. > 8 indicates clinical anxiety.	1. Prior to recall (*n* = 509) 2. A few days after recall (*n* = 34) 3. 3 months after recall (*n* = 34) 4. 1 year after recall (*n* = 31)	1. Mean = 3.4 (SD = 3.) 2. Mean = 4.6 (SD = 3.4) 3. Mean = 4.3 (SD = 4.1) 4. Mean = 3.9 (SD = 4.9)	—	—
Lampic (2002)*, Breast [26]	HADS‐D: scoring same as above. > 8 indicates clinical anxiety.	1. 3 months after recall (*n* = 34) 2. 1 year after recall (*n* = 33)	1. % > 8 = 15% 2. % > 8 = 13%	—	—
McSweeney (2017), Breast [27]	HADS‐D: scoring same as above. 0–7 normal, 8–11 mild depression, 12–15 moderate depression, > 16 severe depression.	Not stated (long‐term survivors) (SnD: *n* = 48; non‐SnD: *n* = 45)	*N* > 11 = 9 (18.8%)	*N* > 11 = 7 (15.5%)	No significant difference (data not shown) [SnD versus non‐SnD: 0.12]
Schou Bredal (2013), Breast [28]	HADS‐D: scoring same as Ekeberg. > 11 indicates clinically relevant depression.	1. At recall (*n* = 80) 2. After results (*n* = 72)	1. Mean = 2.7(SD = 2.9) *n* > 11 = 1.3% 2. Mean = 3.4 (SD = 3.5) *n* > 11 = 6.9%	—	—
Varela‐Moreno (2022), colorectal [35]	HADS‐D: 14 items rated using likert scale ranging from 0–3. > 8 indicated depressive symptoms.	5 years post‐diagnosis (SnD (*n* = 429); non‐SnD = 1851))	*N* > 8 = 80 (19%)	*N* > 8 = 468 (25%)	Non‐SnD significantly higher *p* = 0.005 [SnD versus non‐SnD: −0.22]
Centre for Epidemiologic Studies Depression Scale (CES‐D)					
Miles (2015), colorectal [36]	CES‐D: 10 items, score 0–30, higher scores = worse depression.	5–12 years post‐diagnosis (SnD: *n* = 105, interval: *n* = 98; no screening: *n* = 87)	Mean = 3.97 (SD = 4.14)	Interval: Mean = 5.23 (SD = 4.54) No screening: Mean = 5.36 (SD = 4.72)	No difference between interval and SnD (adjusted); Difference = −0.68 (CI = −2.05–0.69; *p* = 0.33) No difference between interval and no screening (adjusted) Difference = 0.39 (CI = −0.97–0.74; *p* = 0.58) [SnD versus interval: −0.29 SnD versus no screening: −0.32]
Roth (2009), Breast [37]	CES‐D: each symptom rated on 4‐point likert scale ranging from 0–3. Higher scores indicate higher depression, > 16 indicates clinical depression. Total score ranges from 0–60.	1. At diagnosis 2. 1‐year post‐diagnosis (SnD: *n* = 86; non‐SnD: *n* = 75 at both timepoints)	1. Mean = 9.1 (SD = 7.5) 2. Mean = 12.7 (SD = 10.1)	1. Mean = 12.5 (SD = 11.7) 2. Mean = 9.9 (SD = 11.8)	No significant difference at the 2 time points (data not shown); interaction with change over time *p* = 0.032 [SnD versus non‐SnD: 1. −0.35 2. 0.25]
Van den Bergh (2012)^+^, Prostate [31]	CES‐D (§ score range 0–60 (maximum depression), threshold for clinically depressive: ≥ 16).	1. 6‐month post‐diagnosis (*n* = 266) 2. 18‐month post‐diagnosis (*n* = 215)	1. Mean = 7.0 2. Mean = 7.0	—	—
Depression Anxiety and Stress Scale (DASS)—Depression Scale					
Drummond (2016), prostate [34]	DASS (depression scale): 7 items, 4‐point likert scale, 0–3. Total score—0–21. > 10 indicates depression.	2–18 years post‐diagnosis, (SnD: *n* = 1978; clinically detected: *n* = 1331)	*N* ≥ 10 = 14% (CI = 12.2%–15.4%)	*N* ≥ 10 = 21% (CI = 19.2%–22.3%)	Non‐SnD significantly higher *p* < 0.001 (*t*‐test) [SnD versus clinically detected: −0.27]
Self‐reported treatment					
Drummond (2016), prostate [34]	Treatment for depression.	2–18 years post‐diagnosis (SnD: *n* = 1978; clinically detected: *n* = 1331)	*N* = 4%	*N* = 7%	Clinically detected significantly higher *p* < 0.001 (chi‐square test) [SnD versus clinically detected: −0.33]
QUALITY OF LIFE AND HEALTH STATUS					
Short Form‐6, 12 or 36 (SF‐6, SF‐12, SF‐36)					
Booth (2014), prostate [38]	SF‐6D utility value: 6 items, values from 6 items converted into utility score which ranges from scored 0–1 (1 = perfect health, 0 = death).	2011, median of 8.0 years since diagnosis (SnD: *n* = 1587; non‐SnD: *n* = 1706))	Mean = 0.765	Mean = 0.757	No significant difference *p* = 0.072 [Insufficient data to calculate]
Gareen (2014)^+^, lung [30]	SF‐36 physical & mental Component scores (PCS & MCS; score range 0–100 (best health), mean score 50, standard deviation 10; Adults aged 55–64, the median norm PCS is 50.65 & MCS is 55.28. Adults 65–74, the median norm PCS is 46.11 & MCS is 56.11; clinically important change over time is 3–5 points)	1. Baseline (first screening appointment) (*n* = 63) 2. 1‐month post‐screening (*n* = 43) 3. 6‐month post‐screening *(n = 42)*	PCS 1. *M* = 47.3 (SD = 10.9); 2. *M* = 43.7 (SD = 11.4); 3. *M* = 34.5 (SD = 11.8) MCS 1. *M* = 52.6 (SD = 10.3); 2. *M* = 46.7 (SD = 12.8); 3. *M* = 46.3 (SD = 13.2)	—	—
Van den Bergh (2012)^+^, Prostate [31]	SF‐12 physical & mental Component scores (PCS & MCS; score range 0–100 (best health), mean score is 50 in general population.)	1. 6 months post‐diagnosis (*n* = 266) 2. 12–18 months post‐diagnosis (*n* = 215)	PCS 1. *M* = 50.1; 2. *M* = 49.9 MCS 1. *M* = 53.7; 2. *M* = 54.6	—	—
Venderbos (2017), prostate [33]	SF‐12 physical & mental Component scores (PCS & MCS): Domains scored 0–100, 100 indicates best health.	6–8 years post diagnosis (*n* = 70)	PCS *M* = 28.4 (SD = 7.7) MCS *M* = 52.0 (SD = 9.2)	—	—
Vermeer (2020), colorectal [39]	SF‐36: 8 dimensions scored 0–100, higher scores indicate better functioning. Minimal change for clinically important difference is at least 5 points, up to 12.5 points on the Social Functioning Scale.	1. Pre‐colonoscopy (*n* = 68) 2. Post‐colonoscopy (*n* = 60) 3. 6 months (*n* = 49)	Domains Physical functioning 1. *M* = 91; 2. *M* = 90; 3. *M* = 83 Role‐physical 1. *M* = 90; 2. *M* = 83; 3. *M* = 64 Bodily pain 1. *M* = 90; 2. *M* = 91; 3. *M* = 86 Social functioning 1. *M* = 92; 2.*M* = 84; 3. *M* = 85 Mental health 1. *M* = 81; 2. *M* = 75; 3. *M* = 82 Role‐emotional 1. *M* = 91; 2. *M* = 78; 3. *M* = 76 Vitality 1. *M* = 79; 2. *M* = 78; 3. *M* = 70 General health 1. *M* = 76; 2. *M* = 69; *M* = 69	—	—
European Organisation for Research and Treatment of Cancer Quality of Life Questionnaire Core 30 (EORTC‐QLQ‐C30)					
Braun (2020), breast [40]	EORTC QLQ‐C30 v3: Scales scored 0–100, higher scores indicate better outcomes; difference of > 10 points clinically relevant.	Average 6.1 years from diagnosis (SnD: *n* = 346; interval: *n* = 88; clinically detected *n* = 301)	Quality of life *M* = 68.8 (CI = 67.8–70.1) Cognitive functioning *M* = 80.9 (CI = 79.7–82.2) Emotional functioning *M* = 70.2 (CI = 69.0–71.8) Physical functioning *M* = 81.1 (CI = 80.3–82.2) Role functioning *M* = 78.7 (CI = 77.3–80.3) Social functioning *M* = 80.8 (CI = 79.4–82.4)	Interval: Quality of life: *M* = 71.7 (CI = 69.8–74.1) Cognitive functioning: *M* = 77.9 (CI = 75.4–80.5) Emotional functioning Mean = 70.4 (CI = 67.5–73.3) Physical functioning *M* = 82.3 (CI = 80.5–84.3) Role functioning *M* = 75.9 (CI = 73.0–78.9) Social functioning *M* = 78.7 (CI = 76.2–81.6) Clinically detected: Quality of life: *M* = 69.8 (CI = 68.9–71.0) Cognitive functioning: *M* = 80.9 (CI = 79.5–82.4) Emotional functioning: *M* = 68.8 (CI = 67.2–70.2) Physical functioning: *M* = 83.0 (CI = 81.9–84.0) Role functioning: *M* = 79.6 (CI = 78.1–81.3) Social functioning: *M* = 80.1 (CI = 78.7–81.6)	No significant difference (test results not shown) [SnD versus. interval: Quality of life: −0.11 Cognitive functioning: 0.13 Emotional functioning: −0.01 Physical functioning: −0.07 Role functioning: 0.10 Social functioning: 0.08 SnD versus. clinically detected: Quality of life: −0.05 Cognitive functioning: 0.000 Emotional functioning: 0.05 Physical functioning: −0.10 Role functioning: −0.03 Social functioning: 0.03]
Dang (2019)^+^, Colorectal [41]	EORTC QLQ‐C30 version 3 (scales scored 0–100, higher scores indicate better outcomes).	1. Endoscopy: Median 18 months (*n* = 55) 2. Surgery: Median 21 months (*n* = 55)	Global health: *M* = 84.1 (SD = 14.4) Physical functioning: *M* = 91.8 (SD = 11.8) Role functioning: *M* = 91.1 (SD = 18.2) Emotional functioning: *M* = 89.3 (SD = 14.7) Cognitive functioning: *M* = 93.2 (SD = 14.0) Social functioning: *M* = 93.8 (SD = 15.1) Fatigue: *M* = 12.8 (SD = 18.1) Nausea & vomiting: *M* = 3.5 (SD = 10.9) Pain: *M* = 9.9 (SD = 17.3) Dyspnoea: *M* = 9.4 (SD = 18.4) Insomnia: *M* = 13.9 (SD = 21.5) Appetite loss: *M* = 4.2 (SD = 14.3) Constipation: M7.6 (SD = 18.4) Diarrhoea: *M* = 6.4 (SD = 16.6) Financial difficulties: *M* = 2.7 (SD = 15.7)	—	—
European Organisation for Research and Treatment of Cancer Quality of Life Questionnaire, Breast Cancer Module 23 (EORTC QLQ‐BR23) (Breast & Body Image Scale)					
Braun (2020), breast [40]	EORTC QLQ‐BR23 breast & Body image scale (scored 0–100, higher score indicates better outcome).	Average 6.1 years from diagnosis (SnD: *n* = 346; interval: *n* = 88; clinically detected *n* = 301)	*M* = 77.4 (CI = 76.0–79.0)	Interval: *M* = 72.7 (CI = 69.8–75.7) Clinically detected: *M* = 73.8 (CI = 72.3–75.5)	No significant difference (test results not shown) [SnD versus interval: 0.17 SnD versus clinically detected: 0.13]
Expanded Prostate Cancer Index Composite (EPIC)					
Venderbos (2017), prostate [33]	EPIC: 21 items; domains scored 0–100; higher scores indicate better function.	6–8 years post diagnosis (*n* = 70)	Urinary summary: *M* = 85.6 (SD = 14.4) Urinary function: *M* = 80.0 (SD = 19.1) Urinary bother: *M* = 89.9 (SD = 13.1) Urinary incontinence: *M* = 70.1 (SD = 28.8) Urinary irritative: *M* = 95.5 (SD = 7.2) Bowel summary: *M* = 94.6 (SD = 8.0) Bowel function: *M* = 92.6 (SD = 9.2) Bowel bother: *M* = 96.6 (SD = 8.3) Sexual summary: *M* = 34.2 (SD = 14.9) Sexual function: *M* = 14.8 (SD = 17.7) Sexual bother: *M* = 77.8 (SD = 30.2)	—	—
Functional Assessment of Cancer Therapy—Colorectal (FACT‐C)					
Miles (2015), colorectal [36]	FACT‐C total score (score 0–136, higher scores better).	5–12 years post‐diagnosis (SnD: *n* = 94; interval: *n* = 90; no screening: *n* = 82)	*M* = 116.57 (SD = 13.63)	Interval: *M* = 108.26 (SD = 18.37) No screening: *M* = 110.67 (SD = 18.53)	Interval significantly worse than SnD (adjusted); Difference = 6.16 (CI = 0.65–11.66; *p* = 0.03) [SnD versus interval: 0.52 SnD versus no screening: 0.37]
World Health Organization Quality of Life Assessment (WHO‐QOL)					
van der Steeg (2011), breast [32]	WHO‐QOL: Scoring & interpretation not provided.	1. Before diagnosis 2. At diagnosis (*n* = not reported) 3. 1‐month post‐diagnosis 4. 3‐month post‐diagnosis 5. 6‐month post‐diagnosis 6. 12‐month post‐diagnosis (*n* = 152, not reported per timepoint)	1. *M* = 15.6 (SD = 2.4) 2. *M* = 16.0 (SD = 2.4) 3. *M* = 15.4 (SD = 2.6) 4. *M* = 15.7 (SD = 2.5) 5. *M* = 15.7 (SD = 2.5) 6. *M* = 15.9 (SD = 2.5)	—	—
EuroQual five dimension (EQ‐5D)					
Booth (2014), prostate [38]	EQ‐5D‐5L: five items, scores converted into utility value which ranges from 0–1.	2011, median of 8.0 years since diagnosis (SnD: *n* = 1587; non‐SnD: *n* = 1706)	*M* = 0.837	Mean = 0.821	Screening arm significantly better: *p* = 0.017 [Insufficient data to calculate]
Dang (2019)^+^, Colorectal [41]	EQ‐5D‐5L: Scoring as above (Booth) and visual analogue scale (represents patient perspective ‐ score 0 = worst health, 100 = best health).	Median 19.5 months (*n* = 110 (55 endoscopy, 55 surgery))	VAS *M* = 84.7 (SD = 12.1) Descriptive system *M* = 0.88 (SD = 0.11)	—	—
Venderbos (2017), prostate [33]	EQ Visual Analogue scale (scored worst health 0–100 best health)	6–8 years post diagnosis (*n* = 70)	*M* = 78.6 (SD = 15.0)	—	—
15D					
Booth (2014), prostate [38]	15D utility value (scored 0–1, 0 = death, 1 = full health).	2011, median of 8.0 years since diagnosis (SnD: *n* = 1587; non‐SnD: *n* = 1706)	*M* = 0.878	*M* = 0.870	Screening arm significantly better: *p* = 0.031 Screening arm significantly better longitudinally from 1998–2011. Difference = 0.16, *p* < 0.001 [Insufficient data to calculate]
GENERIC PSYCHOLOGICAL DISTRESS					
General health questionnaire (GHQ)					
Barrett* (2014), ovarian [29]	GHQ‐12: 12 items. Total score 1–12. Scores ≥ 4 = psychological morbidity.	0. Screening appointment 1. 6 weeks post‐surgery 2. 6 months post‐surgery	Comparison with screening appointment 1. OR (score ≥ 4) = 16.2 (CI = 9.19–28.54) 2. OR (score ≥ 4) = 3.32 (CI = 1.91–5.77)	—	—
Ellman (1989), breast [42]	GHQ: 12 items. Total score 1–12. Scores > 4 = psychological morbidity.	3 months after clinic attendance (SnD: *n* = 18; non‐SnD: *n* = 19)	Score > 4 = 9/18 (50%)	Not presented for symptomatic cancer alone	—
Menon* (2023), ovarian [43]	GHQ: 12 items. Total score 1–12. Scores ≥ 4 = psychological morbidity.	0. Screening appointment (SnD: *n* = 1042; Non‐SnD: *n* = 1016) 1. 6 weeks post‐surgery (*n* = not reported) 2. 6 months post‐surgery (*n* = not reported)	Comparison with screening appointment 1. OR = 16.2 (CI = 9.19–28.54) 2. OR = 3.31 (CI = 1.91–5.77)	—	—
Structured psychological interview & categorization of symptoms using the diagnostic and statistical manual of mental disorders (DSM)‐III					
Burgess (2002), breast [44]	Anxiety or depressive episode.	Interviews at 5 and 18 months post‐diagnosis	*N* = 24/52 (46%)	*N* = 37/80 (46%)	No significant difference *p* = 1.0 (Fisher's Exact test) [SnD versus non‐SnD ≤ −0.01]
Haddad (1994), breast [45]	Anxiety or depressive symptoms.	1. 1–2 months post‐diagnosis 2. 12–13 months post‐diagnosis 3. 1–14 months post‐diagnosis (total) (SnD: *n* = 82; non‐SnD: *n* = 213)	1. *N* = 7 (9%) 2. *N* = 3 (4%) 3. *N* = 13 (16%)	1. *N* = 20 (9%) 2. *N* = 14 (7%) 3. *N* = 48 (23%)	No significant difference 1. *p* = 0.923; 2.*p* = 0.307; 3. *p* = 0.216 [SnD versus non‐SnD: 1. −0.06; 2. −0.34; 3. −0.24]
Medical record data					
Lu (2019), Cervical [46]	Presence of stress reaction and adjustment, depression or anxiety disorder.	One year before cancer diagnosis onward; focus on disorders within 1‐year before/after diagnosis. (SnD: *n* = 1241; non‐SnD: *n* = 3004)	*N* = 9.6%	*N* = 10%	No significant difference OR = 1.13 (0.88–1.44) [SnD versus non‐SnD: −0.03]
Politi (2020), breast [47]	Medical record codes for fatigue, anxiety or depression.	Up to 16 years post diagnosis (SnD: *n* = 134; non‐SnD: *n* = 77	*N* = 16 (11.94%)	*N* = 16 (20.78%)	No significant difference *p* = 0.085 [SnD versus non‐SnD: −0.36]
Impact of Events Scale (IES)					
Gibbons (2017), breast [23]	IES‐ 15 items, 4‐point likert scale; scored from 0 (not at all), to 5 (often). Items are summed to give IES score. Higher scores indicate higher levels of stress.	12 months post‐diagnosis (At both timepoints‐ SnD: *n* = 92; symptomatically: *n* = 129)	*M* = 25.06 (SD = 16.91) *N* > 33 = 17 (33.3%)	*M* = 16.5 (SD = 15.56) *N* > 33 = 15 (13.5%)	SnD significantly higher: t (159) = 3.12; *p* = 0.002 [SnD versus symptomatic: 0.53]
Depression Anxiety Stress Scales (DASS)					
Drummond (2016), prostate [34]	DASS (stress scale)‐ 21 question version ‐ 4‐point likert scale from 0 ‘did not apply to me at all’ to 3 ‘Applied to me very much, or most of the time’. Scores were doubled for analysis. Respondents categorized as being stressed if scored ≥ 15.	2–18 years post prostate cancer diagnosis (SnD: *n* = 1978; clinically detected: *n* = 1331)	*N* ≥ 15 = 9% (CI = 7.9%–9.7%)	*N* ≥ 15 = 14% (CI = 12.8%–15.1%)	Non‐SnD significantly higher *p* < 0.001 (chi‐square test) [SnD versus clinically detected: −0.28]
Cortisol					
Gustafsson (1995), prostate [48]	Serum cortisol (difficult to define what normal serum cortisol levels are).	1. Screening (all) (*n* = 100) 2. Biopsy 2 weeks after screening (includes all participants) (*n* = 307) 3. 4 weeks after screening (cancer only) (*n* = 34) 4. 16 weeks after screening (cancer only) (*n* = 33)	1. *M* = 478.8 (CI = 450.7–506.9) 2. *M* = 495.9 (CI = 478.2–513.6) 3. *M* = 479.3 (CI = 429.6–529.0) 4. *M* = 448.2 (CI = 389.2–507.2)	—	—
CANCER‐SPECIFIC PSYCHOLOGICAL DISTRESS					
Cancer Worry Scale					
Dang (2019)^+^, colorectal [41]	Cancer worry scale: 4 items on 10‐point scales, summed for the total score (0–40). Higher scores = more fear of cancer recurrence.	Median 19.5 months (*n* = 110 (55 endoscopy, 55 surgery))	*M* = 8.7 (SD = 6.6)	—	—
Field (2016), lung [22]	Cancer worry scale: 6 items on 4‐point scales, summed for total score (6–24). Score > 12.5 = clinically relevant distress.	1. Baseline before CT scan (people later invited to screening *n* = 2018) 2. Two weeks after initial scan (positive referrals, *n* = 48) 3. 10–27 months later (true positives, *n* = 23)	1. *M* = 8.75 (% > 12.5 = 10%) 2. *M* = 11.88 (CI = 11.10–12.72) 3. *M* = 9.01 (CI = 8.16–9.96)	—	—
Vermeer (2020), colorectal [39]	Cancer worry scale: 6‐Items on 4‐point scales, total score 6–24. Score ≥ 7 low, ≥ 10 high.	1. Pre‐colonoscopy (*n* = 60) 2. Post‐colonoscopy (*n* = 60) 3. 6 months (*n* = 50)	1. Median = 9.5 2. Median = 10.5 3. Median = 9.5	—	—
Cancer Distress Thermometer					
Fortin (2023), breast [49]	Distress thermometer (scoring: 0 = no distress, 10 = extreme distress; ≥ 4 clinical distress).	During treatment, but asked to retrospectively consider: 1. Worst breast cancer related moment 2. Last 7 days (At both timepoints‐ SnD: *n* = 7; non‐SnD *n* = 11)	1. *M* = 6.64 (SD = 1.80) 2. *M* = 1.86 (SD = 1.87)	1. *M* = 7.27 (SD = 2.08) 2. *M* = 3.36 (SD = 2.17)	—
Yang (2023), breast [50]	Adapted distress thermometer (scoring: Scale of 0 (no distress) to 10 (extreme distress) in four domains (emotional, social, health, and practical).	At diagnosis (n is reported for each domain)	Domains Emotional concern (*n* = 397) High (> 6) = 167 (42.1%) Median (IQR) = 5 (3–7) Social concern (*n* = 387) High (> 6) = 56 (14.5%) Median (IQR) = 1 (0–4) Health concern (*n* = 384) High (> 6) = 166 (43.2%) Median (IQR) = 5 (3–7) Practical concern (*n* = 375) High (> 6) = 80 (21.3%) Median (IQR) = 2 (0–5)	Domains Emotional concern (*n* = 340): High (> 6) = 182 (53.5%); median (IQR) = 6 (4–8) Social concern (*n* = 304) High (> 6) = 56 (25.8%) Median (IQR) = 2 (0–6) Health concern (*n* = 327) High (> 6) = 182 (55.7%) Median (IQR) = 6 (3–8) Practical concern (*n* = 330): High (> 6) = 104 (31.5%); median (IQR) = 3 (1–6)	Symptomatic‐detection significantly higher in each domain. Domains Emotional concern: High (> 6) *p* = 0.002; median *p* = 0.002 Social concern: High (> 6) *p* < 0.001; median *p* = 0.002 Health concern: High (> 6) *p* = 0.002; median *p* = 0.001 Practical concern: High (> 6) *p* = 0.002; median *p* < 0.0001 [SnD versus. symptomatic: Emotional concern: −0.25 Social concern: −0.16 Health concern: −0.28 Practical concern:−0.29]
Psychological Consequences of Screening Questionnaire (PCQ)					
Vermeer (2020), colorectal [39]	PCQ negative scale: 12 items scored 0–3. Total score 0–36, higher score = more dysfunction.	1. Pre‐colonoscopy (*n* = 60) 2. Post‐colonoscopy (*n* = 60) 3. 6 months (*n* = 50)	1. Median = 5.0 2. Median = 9.0 3. Median = 5.0	—	—
Consequences of Screening Questionnaire—Breast (COS‐B)					
Solbjør (2018), breast [51]	COS‐B (Part‐I: items scored 0–3. Higher scores = worse outcomes. Scales have the following number of items: Felt less attractive (1), mind off things (1), anxiety (6), sense of dejection (6), negative impact on behaviour (7), sleep (4), breast self‐examination (2), sexuality (2). Part‐II: Items scored 0–3. Scales have the following number of items: Existential values (6), social network (3), inner calm (2). Higher values = more change.	1. At screening result (*n* = 28) 2. 1 month (*n* = 22) 3. 6 months (*n* = 22)	Domains Dejection: 1. *M* = 4.2 (CI = 2.3–6.1); 2. *M* = 5.7 (CI = 3.6–7.8; 3. *M* = 3.4 (CI = 1.5–5.2) Anxiety: 1. *M* = 3.2 (CI = 1.5–5.0); 2. *M* = 4.9 (CI = 3.0–6.7); 3. *M* = 3.2 (CI = 1.5–4.9) Behaviour: 1. *M* = 2.6 (CI = 1.1–4.0); 2. *M* = 4.1 (CI = 2.4–5.7); 3. *M* = 4 (CI = 1.6–6.3) Sleep: 1. *M* = 1.6 (CI = 0.7–2.5); 2. *M* = 2.6 (CI = 1.5–3.9); 3. *M* = 2.5 (CI = 1.2–3.8) Breast self‐exam: 1. *M* = 2.7 (CI = 2–3.4); 2. *M* = 2.4 (CI = 1.7–3); 3. *M* = 1.9 (CI = 1.4–2.4) Sexuality: 1. *M* = 1.1 (CI = 0.3–1.8); 2. *M* = 2.3 (CI = 1.4–3.2); 3. *M* = 2.7 (CI = 1.5–3.8) Attractive: 1. *M* = 0.0 (CI = 3–0.1); 2. *M* = 0.2 (CI = 0–0.5); 3. *M* = 0.5 (CI = 0.1–0.8) Mind off things: 1. *M* = 0.7 (CI = 0.3–1); 2. *M* = 1.2 (CI = 0.9–1.6); 3. *M* = 0.5 (CI = 0.3–0.8) Existential: 1. M = Not applicable; 2. *M* = 4.4 (CI = 3.1–5.4); 3. *M* = 3.5 (CI = 2.6–4.4) Social network: 1. M = Not applicable; 2. *M* = 1.2 (CI = 0.7–1.6); 3. *M* = 1.4 (CI = 0.8–2) Inner calm: 1. M = not applicable; 2. *M* = 1.8 (CI = 1.2–2.4); 3. Mn = 1.6 (CI = 0.4–2.9)	—	—
Cancer‐related distress					
Gibbons (2016), breast [24]	4 questions adapted from previous research. Total score 4–20, higher scores = greater distress.	After dx/before tx (*n* = 94)	Mean = 14.1 (SD = 4.10) *N* > 11 = 6 (6.5%)	—	—

*Note:* Menon 2023 and Orbell 2008 collected but did not descriptively report anxiety data using the STAI so they are not included in the anxiety section of the table. Menon 2023 collected but did not report results for the FACT‐O, so this is not included in the quality of life section of the table. *Papers present data from the same study; +papers present average mean data from treatment or diagnostic test groups (see supporting information for means reported in the original paper).

Abbreviations: M = Mean; SD=Standard deviation; SnD = Screen Detected cancer group.

Papers examined the impact of breast (*n* = 18), prostate (*n* = 5), colorectal (*n* = 5), lung (*n* = 2), ovarian (*n* = 2) and cervical (*n* = 1) cancer screening. Fifteen papers presented psychological outcomes by detection route. Twenty‐nine studies collected self‐reported questionnaire data using 31 different measures. Two studies conducted structured psychological interviews, two used medical record data and one collected biological samples reporting cortisol levels.

A broad range of psychological outcomes were reported including anxiety (reported by *n* = 17 papers), depression (*n* = 14), quality of life or health status (*n* = 10), generic psychological distress (*n* = 10), and cancer‐specific psychological distress (*n* = 8). Nine studies assessed a range of other psychological outcomes.

Quality scores were 100% for 4 papers, 80% for 20 papers, 60% for 6 papers and 40% for 3 papers. The quality criteria of each paper are reported in the Supporting Information.

### 3.2 Anxiety

Fifteen studies (across 17 papers) reported anxiety (see Table 2), with eight studies using the Hospital Anxiety and Depression Scale (HADS‐A) [20, 21, 22, 23, 24, 25, 26, 27], six using the State Trait Anxiety Inventory (STAI) [29, 30, 31, 32, 33, 53], and one using the Depression Anxiety Stress Scale (DASS‐21) [34].

#### 3.2.1 Anxiety in Patients With Screen‐Detected Cancer

**FIGURE 2:**
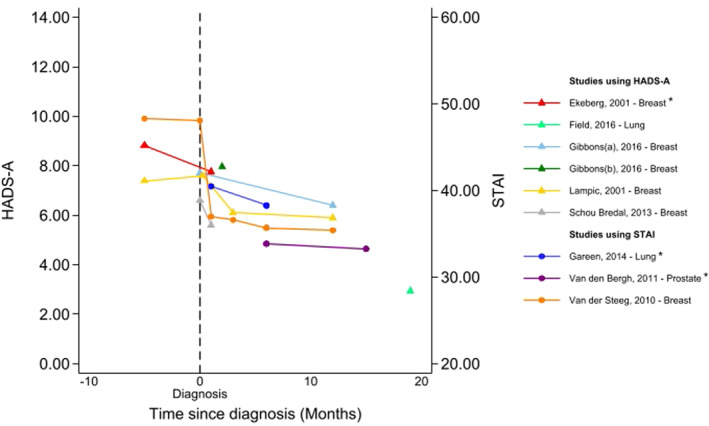
Anxiety among participants with screen‐detected cancer reported over time using the STAI or HADS‐A. *Anxiety scores were reported for sub‐groups of those with screen‐detected cancer (e.g., by treatment) so an overall mean has been calculated for presentation. Where cut‐offs for anxiety were reported, these ranged from > 8 to > 11 (for HADS‐A) and were > 44 (for the STAI); Data beyond 24 months from diagnosis are not presented here but can be seen in supporting Information.

Across studies, 13%–44% of patients with screen‐detected cancer met a study‐defined threshold for anxiety (see Table 2). The level (e.g., cut‐off score) and meaning of the threshold (e.g., clinically relevant, borderline case) varied by study. Studies tended to report slightly elevated anxiety levels around the time of the screening result and diagnostic tests, which declined over time (see Figure 2). Time points used to report longer‐term anxiety varied greatly (from 12 months, up to 8 years post screen‐detected diagnosis).

#### 3.2.2 Anxiety by Cancer Detection Route

Four studies examined the difference in anxiety between patients with screen‐detected cancer and those with non‐screen‐detected cancer [21, 23, 27, 34].

Three of these were in breast screening and used the HADS‐A, finding no significant difference by detection route [21, 23, 27]. Of these, one collected data at recall mammogram following an abnormal screen and 4 weeks post‐diagnosis [21], one collected data cross‐sectionally among ‘long‐term survivors’ [27] and the last collected data at diagnosis and 12 months later [23]. One of these reported mean scores by detection route over time [23], presented in Figure 4.

One retrospective prostate screening study that assessed anxiety using the DASS, surveyed patients 2–18 years post‐diagnosis [34]. Anxiety in the last week was significantly less common among patients with screen‐detected cancer compared to patients with non‐screen‐detected cancer, controlling for clinical and socio‐demographic factors.

### 3.3 Depression

Thirteen studies reported depression in 14 papers. Ten papers reported data from nine studies using the HADS‐D [20, 21, 22, 23, 24, 25, 26, 27, 28, 35], three used the Centre for Epidemiological Studies—Depression Scale (CES‐D) [31, 36, 37], one used the DASS‐21 as well as self‐reported treatment for depression [34] (see Table 2 for details).

#### 3.3.1 Depression in Patients With Screen‐Detected Cancer

**FIGURE 3:**
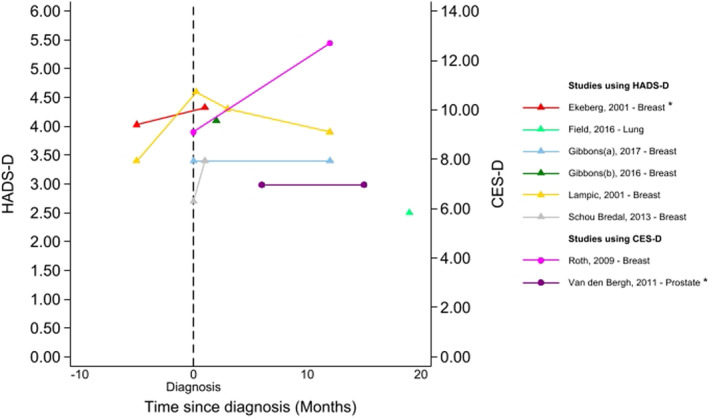
Depression among participants with screen‐detected cancer reported over time using the HADS‐D or CES‐D. *Depression scores were reported for sub‐groups of those with screen‐detected cancer (e.g., by treatment) so an overall mean was calculated for presentation. Where cut‐offs for depression were reported, these ranged from > 8 to > 14 (for HADS‐D) and > 16 (for CES‐D); Data beyond 24 months from diagnosis are not presented here but can be seen in supporting Information.

Studies reported that 1%–19% of patients with screen‐detected cancer met a study‐defined threshold for depression. Thresholds varied across studies. Levels of depression seemed to stay relatively stable over time (Figure 3). Longitudinal studies collected data from the time of screening test up to 18 years post‐diagnosis.

**FIGURE 4:**
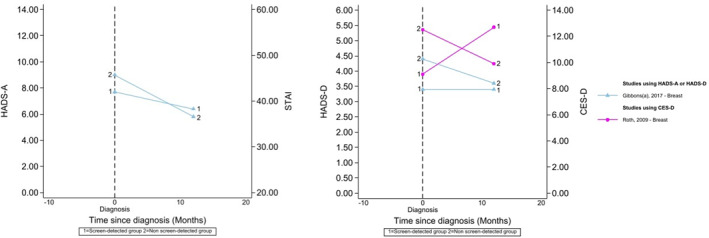
Anxiety (using HADS‐A or STAI) and Depression (using HADS‐D or CES‐D) from studies that reported means by detection route over time. Where cut‐offs for anxiety were reported, these ranged from > 8 to > 11 (for HADS‐A); > 44 (for the STAI); > 8 to > 14 (for HADS‐D) and > 16 (for CES‐D); Data beyond 24 months from diagnosis are not presented here but can be seen in supporting Information.

#### 3.3.2 Depression by Cancer Detection Route

Seven studies examined the difference in depression between patients with screen‐detected and non‐screen‐detected cancer [21, 23, 27, 34, 35, 36, 37]. Two of these reported mean scores by detection route over time using the HADS‐D/CES‐D (see Figure 4) [23, 37].

Five studies found no significant difference in depression by detection route [21, 23, 27, 36, 37]. This included four breast screening studies with data collected cross‐sectionally among ‘long‐term survivors’ [21, 27], or at diagnosis with 12‐month follow‐up [23, 37]. One colorectal screening study collected data 5–12 years post‐diagnosis [36].

Two cross‐sectional studies found depression was less common in patients with screen‐detected cancers compared to non‐screen‐detected cancers. This included one prostate screening study collecting data 2–18 years after diagnosis [34] and one colorectal screening study collecting data five years post‐diagnosis [35]. However, effect sizes were small (*d* = −0.27 and −0.22, respectively).

### 3.4 Quality of Life (QoL) and Health Status

Ten papers reported one or more QoL or health status outcome. Five studies used the Medical Outcome Study Short Form (SF‐6, SF‐12, SF‐36) [30, 31, 33, 38, 39], two studies used the EORTC QLQ‐C30 [40, 41], one of which also used part of the EORTC QLQ‐BR23 [40], one study used the Expanded Prostate Cancer Index Composite questionnaire (EPIC) [33], one study used the Functional Assessment of Cancer Therapy ‐ Colorectal (FACT‐C) [36], and one study used the World Health Organization Quality of Life scale (WHOQOL) [32]. Three studies reported health status, all of which used the EuroQol questionnaire (EQ‐5D) [33, 38, 41]. One study additionally used the 15D questionnaire [33].

#### 3.4.1 QoL and Health Status in Patients With Screen‐Detected Cancer

Descriptive results can be found in Table 2 for patients with screen‐detected cancer. There were no clear patterns in QoL or health status over time.

#### 3.4.2 QoL and Health Status in Screen‐Detected Cancer Compared to Other Routes

Three studies compared QoL or health status by detection mode, with mixed findings [36, 38, 40]. One prostate cancer study [38] found men with screen‐detected cancer had significantly better QoL than men with non‐screen‐detected cancer 11–14 years after study enrolment (using EQ‐5D and 15D). However, the authors found no significant difference when using the SF‐6D in the same sample. In a colorectal screening study that collected data 5–12 years after cancer diagnosis, patients with non‐screen‐detected cancers had worse QoL than patients with screen‐detected cancers after controlling for co‐variates [36]. This difference had a small effect size (*d* = 0.29). One breast screening study showed no significant difference in QoL by cancer detection route after adjusting for covariates [40].

### 3.5 Generic Psychological Distress

Ten papers reported generic psychological distress from nine studies. Three papers used the General Health Questionnaire (GHQ‐28) [29, 42, 43], two used structured psychological interviews [44, 45], two used medical record data [46, 47], one used the Impact of Events Scale (IES) [23], one used the DASS‐21 [34], and one used cortisol level [48].

#### 3.5.1 Generic Distress in Patients With Screen‐Detected Cancer

One study in breast cancer found that 50% of screen‐detected cancer patients met the threshold for psychological morbidity (GHQ score > 4) 3 months after mammography [42]. Using structured psychological interviews, Haddad et al., found 9% of patients with breast cancer reported anxiety or depressive symptoms in the 12 months following diagnosis [45], whereas Burgess et al. found 46% of screen‐detected breast cancer patients self‐reported having an episode of anxiety or depression in the year following cancer diagnosis [44]. Another, with ovarian cancer patients, reported that distress (GHQ score ≥ 4) peaked 6 weeks after surgery but reduced at 6 months (reported in two papers) [29, 43].

Two studies looked at medical record data. One reported that 11.9% of women had fatigue, anxiety or depression diagnosed in the 16 years following a screen‐detected breast cancer diagnosis [47]. Another reported a similar proportion (9.6%) among patients with screen‐detected cervical cancer, however this included diagnoses in the year before and the year after diagnosis [46].

A breast screening study found about 33% of women had clinically relevant levels of distress 12 months after cancer diagnosis (using the IES) [23], while a prostate screening study found that only 9% of participants experienced distress 2–18 years after diagnosis (using the DASS stress scale) [34].

One study used cortisol levels among patients with prostate cancer [48]. Levels peaked at the time of biopsy (2‐week post screening), declining by 4‐ and 16‐week, but these changes were not significantly different and no threshold for clinically relevant distress was provided.

#### 3.5.2 Generic Psychological Distress by Detection Route

Six papers examined the difference in generic psychological distress by cancer detection route [23, 34, 44, 45, 46, 47].

Drummond et al. found a lower proportion of patients with screen‐detected prostate cancer were ‘stressed’ compared to those with non‐screen‐detected cancer, 2–18 years post‐diagnosis (using the DASS). However, the size of the difference was small (*d* = −0.28) [34]. In contrast, Gibbons et al. found that women with screen‐detected breast cancer had significantly higher distress 12‐month post‐diagnosis compared to non‐screen‐detected patients on the IES with a medium effect size (*d* = 0.53) [23].

The remaining studies that considered a difference by detection route used structured psychological interviews or medical record data and found no significant difference [44, 45, 46, 47].

### 3.6 Cancer‐Specific Distress

Eight papers reported on cancer‐specific distress. Three studies used the Cancer Worry Scale [22, 39, 41], two used cancer distress thermometers [49, 50], one used the Psychological Consequences of Screening (PCQ) questionnaire [39], one used the Consequences of Screening ‐ Breast Cancer questionnaire (COS‐BC) [51], and one assessed cancer‐related distress with study‐specific items [24].

#### 3.6.1 Cancer‐Specific Distress in Patients With Screen‐Detected Cancer

Two studies using the 6‐item Cancer Worry Scale longitudinally in lung and colorectal studies found that cancer worry peaked after receiving screening test results but before diagnosis [22, 39]. Both studies assessed cancer worry before screening, after screening and 6–27 months after diagnosis. The peak reached the threshold for ‘high’ worry in colorectal screening but did not reach clinical relevance in lung screening; however, the clinical relevance thresholds were different in the two studies (> 12.5 and ≥ 10 respectively). A third study using the Cancer Worry Scale in patients with screen‐detected colorectal cancer 18–21 months post diagnosis found mean worry was 8.7 (possible range 0–40), but no threshold for clinical relevance was provided [41].

Two breast screening studies used distress thermometers. One study collecting data during treatment found that participants' retrospective rating of distress at their ‘worst breast cancer related moment’ met the threshold for clinical distress (≥ 4) but distress in the last 7 days did not (screen‐detected data provided by the authors) [49]. Another study collecting data at diagnosis found that median scores in specific domains (emotional, social, health and practical concern) did not meet thresholds for ‘high’ distress (> 6), but that over 40% of participants met the thresholds at an individual level in the emotional and health domains [50].

Studies looking at the psychological consequences of screen‐detected breast and colorectal cancer (using the PCQ and COS‐BC) found generally low levels of concern across domains [39, 51]. In both studies, scores tended to be worse after the screening result compared to before, at the screening test or 6 months later. Another study reported that levels of distress were infrequent (6.5%) in the period between diagnosis and treatment for patients with screen‐detected breast cancer [24].

#### 3.6.2 Cancer‐Specific Distress by Cancer Detection Route

Yang et al. found that patients with screen‐detected cancers had significantly lower concerns at diagnosis for each of the domains of the distress thermometer compared to patients with non‐screen‐detected breast cancer (*d* = −0.16 to −0.29) [50].

### 3.7 Other Psychological Outcomes

Other psychological outcomes included illness representations [24, 53], coping [24, 53], decisional regret [22, 39], perceived recurrence risk [37], life values [26], personality [32], perceived diagnostic delay [36], trust in screening results [36] and sleep disturbance [48]. Descriptive results for patients with screen‐detected cancer can be found in the supporting information.

#### 3.7.1 Other Psychological Outcomes by Detection Route

Two studies compared three unique outcomes by detection route. Miles, et al. examined perceived diagnostic delay and trust in screening results 5–12 years after a colorectal cancer diagnosis. The authors found that participants with a screen‐detected cancer perceived significantly less delay in diagnosis and had significantly more trust in the screening test than participants with non‐screen‐detected cancers with medium effect sizes (*d* = 0.54 and −1.36, respectively) [36]. Roth, et al. used a single question to examine perceived risk of recurrence 1 year after a breast cancer diagnosis and found no significant difference by detection route [37].

## 4 Discussion

Overall, the studies included in this review suggest that people who have a cancer diagnosed following an abnormal screening result tend to experience a small or moderate increase in adverse psychological outcomes. This was seen across most outcomes included in this review but especially for anxiety, cancer‐specific distress and general distress. A substantial proportion of patients with screen‐detected cancer in most studies did experience clinically relevant adverse effects at some point in their journey, suggesting the potential for adverse psychological impact should be carefully considered. When looking at the patterns of adverse psychological outcomes at different time points, these tended to peak in the period between the abnormal screening test result and cancer diagnosis and decrease over time.

Our findings for patients with screen‐detected cancer were in line with previous reviews that have looked at the psychological impact of cancer screening over time [12, 54]. We have confirmed that a screen‐detected cancer diagnosis can negatively impact psychological outcomes but that this impact declines over time. The evidence is somewhat variable, but studies have found similar patterns among patients with cancer detected through any route [55, 56, 57]. Some of these studies also found the main predictor of high or persistent distress was baseline distress or a previous psychological condition [56, 57]. Our findings differ from the review by Cotter et al., [16] which suggests there is moderate psychological harm from screen‐detected prostate cancer. However, that review compared patients with cancer to non‐cancer populations, and did not report on the frequency or severity of outcomes.

All studies, except one, presenting psychological outcomes by cancer detection route showed either no significant difference between groups, or that patients with screen‐detected cancers fared better. Outcomes were better for patients with screen‐detected cancer across a range of constructs including anxiety, depression, cancer‐specific distress, generic distress, quality of life, perceived diagnostic delay and trust in screening tests. The differences were small but in the same direction across many studies. A recent paper, published after our search was carried out, reported statistically significant differences in several quality‐of‐life outcomes between patients with screen‐detected (*n* = 691) and those with clinically‐detected (*n* = 480) breast cancer, with better outcomes in those who had a screen‐detected cancer [58]. As with our findings, the differences were small, and the authors described the clinical relevance as limited. However, at a population level, these small differences could have a notable impact on health and health care utilisation. This is reassuring given the increase in focus on early cancer detection with new technologies expanding the potential to increase the number of screen‐detected cancers [59]. While caution needs to be taken in interpreting the results due to the heterogeneity in study design, this suggests that increasing the proportion of patients diagnosed with cancer through screening should not exacerbate poor psychological outcomes for those who go on to have a cancer diagnosed.

Despite qualitative literature suggesting that there may be psychological benefit from detecting cancer through screening [9, 10, 11], few studies assessed positive outcomes. Where positive outcomes were measured using questionnaires like the PCQ, the results were presented among a range of scales, making the findings difficult to interpret. To understand if increasing the number of screen‐detected versus non‐screen‐detected cancers could be psychologically beneficial, future studies should capture positive outcomes using a more rigorous, systematic approach.

### 4.1 Strengths and Limitations

Interpretation and comparison of the results within and across studies was challenging and limited by the heterogeneity of the studies. The studies included a number of different screening modalities, some of which have only been used in the trial context and others that are no longer recommended. Extrapolating psychological outcomes between screening tests may not always be appropriate and future research should continue to consider the psychological impact of being diagnosed with cancer via emerging screening technologies. Studies measured a broad range of psychological outcomes, used a variety of measures for each outcome, collected data at different time points, analysed and presented the data using varying statistical approaches, and interpreted the results in different ways (e.g., norm data comparisons, thresholds for clinical importance, minimally important differences etc.) or omitted this information completely. Most studies also used generic outcome measures which may not be sensitive enough to capture small differences or capture the right constructs for the cancer screening context. Similar challenges have been highlighted in quality‐of‐life research among cancer patients and coordinated efforts are underway to standardise data collection and analysis approaches [60]. Similar standards should be adopted in the cancer screening field to enable better interpretation and comparison of results.

Most studies included were of good quality, however, two papers only met 40 percent of the quality criteria. As we could not conduct a meta‐analysis, we did not adjust for quality in presenting the findings so we suggest study quality is considered when interpreting the findings.

As mentioned above, the narrative summary was limited by the heterogeneity in the studies and reporting. We calculated Cohen's *d* effect sizes to facilitate comparisons, however, these calculations rely on measures of means and standard deviations, and so single observations could have dramatically affected values. In addition, Cohen's *d* assumes normal distribution and homogeneity of variance of the data, which was rarely reported in papers, and thus could not be guaranteed. Effect size calculations may also have been skewed by confounding factors like time since diagnosis.

Most papers reported on breast cancer (*n* = 17), followed by prostate (*n* = 5) and colorectal cancer (*n* = 5). This may mean the results are most applicable to screen‐detected breast cancer patients compared to other cancer types. Of the screening programmes in the UK, there was only one study conducted in cervical cancer and one in lung cancer. This may be because cervical screening is a preventative programme, meaning few people are diagnosed with cervical cancer through screening, and because lung screening has only recently been introduced.

### 4.2 Clinical Implications

It is reassuring to see that overall, the negative psychological impact of cancer screening is limited and adverse outcomes reduce over time. However, studies showed that a substantial minority of screening participants do experience clinically relevant negative outcomes, particularly anxiety, at the time of screening and cancer diagnosis. A variety of interventions have been developed for different screening programmes and cancer patients to help mitigate these outcomes. Further work should be done to understand how best to support patients in the time between screening, diagnosis and treatment, when anxiety may be particularly high.

## 5 Conclusions

Receiving a cancer diagnosis following screening may have a small to moderate negative impact on psychological outcomes, particularly anxiety, cancer worry and general distress. Longitudinally, adverse psychological outcomes tended to peak between the screening tests and cancer diagnosis and decrease over time. While there were some statistical differences in psychological outcomes between those with screen‐detected and non‐screen‐detected cancers, the evidence of a clinically relevant difference by detection mode is limited. Findings were limited by the heterogeneity of the studies in relation to outcomes, data collection time points, analyses and reporting. Future work should consider how best to support patients across the pathway from receiving a screening result to a cancer diagnosis and treatment, and should take into account the impact that their route to diagnosis may have had.

## Author Contributions

**Emma Lidington:** formal analysis, investigation, data curation, writing – original draft, writing – review and editing, visualisation, project administration. **Divya Ragupathy:** formal analysis, investigation, data curation, writing – original draft, writing – review and editing, visualisation, project administration. **Ninian Schmeising‐Barnes:** formal analysis, investigation, data curation, writing – review and editing, visualisation. **Amanda Dibden:** formal analysis, investigation, writing – review and editing, visualisation. **Jo Waller:** conceptualisation, methodology, writing – review and editing, supervision. **Laura Marlow:** conceptualisation, methodology, writing – review and editing, supervision.

## Funding

Emma Lidington was funded by Cancer Research UK core funding for the Queen Mary University of London Cancer Prevention Trials Unit [CTUQQR‐Dec22/100005].

## Conflicts of Interest

JW reports research income from GRAIL Bio UK Ltd, which funded the full salaries of LAVM and NSB through a contract with King's College London/Queen Mary University of London while this work was being carried out.

## Supporting information


Supporting Information S1

## References

[1] N. Hawkes , “Cancer Survival Data Emphasise Importance of Early Diagnosis,” BMJ 364 (2019): l408, 10.1136/bmj.l408. 30683652

[2] A. Barratt , P. Mannes , L. Irwig , L. Trevena , J. Craig , and L. Rychetnik , “Cancer Screening,” Journal of Epidemiology & Community Health 56, no. 12 (2002): 899–902, 10.1136/jech.56.12.899. 12461108 PMC1756972

[3] Cancer Research UK , Screening for Cancer, https://www.cancerresearchuk.org/about‐cancer/spot‐cancer‐early/screening.

[4] A. Stang and K.‐H. Jöckel , “The Impact of Cancer Screening on All‐Cause Mortality,” Deutsches Ärzteblatt international 115, no. 29–30 (2018): 481–486, 10.3238/arztebl.2018.0481. 30135006 PMC6121088

[5] H. D. Nelson , R. Fu , A. Cantor , M. Pappas , M. Daeges , and L. Humphrey , “Effectiveness of Breast Cancer Screening: Systematic Review and Meta‐Analysis to Update the 2009 U.S. Preventive Services Task Force Recommendation,” Annals of Internal Medicine 164, no. 4 (2016): 244–255, 10.7326/M15-0969. 26756588

[6] B. Heleno , M. F. Thomsen , D. S. Rodrigues , K. J. Jorgensen , and J. Brodersen , “Quantification of Harms in Cancer Screening Trials: Literature Review,” BMJ 347, no. 1 (2013): f5334, 10.1136/bmj.f5334. 24041703 PMC4793399

[7] L. Davies , D. B. Petitti , L. Martin , M. Woo , and J. S. Lin , “Defining, Estimating, and Communicating Overdiagnosis in Cancer Screening,” Annals of Internal Medicine 169, no. 1 (2018): 36–43, 10.7326/M18-0694. 29946705

[8] R. P. Harris , S. L. Sheridan , C. L. Lewis , et al., “The Harms of Screening,” JAMA Internal Medicine 174, no. 2 (2014): 281, 10.1001/jamainternmed.2013.12745. 24322781

[9] N. Moshina , R. S. Falk , E. Botteri , et al., “Quality of Life Among Women With Symptomatic, Screen‐Detected, and Interval Breast Cancer, and for Women Without Breast Cancer: A Retrospective Cross‐Sectional Study From Norway,” Quality of Life Research 31, no. 4 (2022): 1057–1068, 10.1007/s11136-021-03017-7. 34698976 PMC8547129

[10] J. Ostero , V. Siersma , and J. Brodersen , “Breast Cancer Screening Implementation and Reassurance,” European Journal of Public Health 24, no. 2 (2014): 258–263, 10.1093/eurpub/ckt074. 23788014

[11] S. B. Cantor , R. J. Volk , A. R. Cass , J. Gilani , and S. J. Spann , “Psychological Benefits of Prostate Cancer Screening: The Role of Reassurance,” Health Expectations 5, no. 2 (2002): 104–113, 10.1046/j.1369-6513.2002.00166.x. 12031051 PMC5060134

[12] A. Kim , K. C. Chung , C. Keir , and D. L. Patrick , “Patient‐Reported Outcomes Associated With Cancer Screening: A Systematic Review,” BMC Cancer 22, no. 1 (2022): 223, 10.1186/s12885-022-09261-5. 35232405 PMC8886782

[13] K. Pickles , J. Hersch , B. Nickel , J. S. Vaidya , K. McCaffery , and A. Barratt , “Effects of Awareness of Breast Cancer Overdiagnosis Among Women With Screen‐Detected or Incidentally Found Breast Cancer: A Qualitative Interview Study,” BMJ Open 12, no. 6 (2022): e061211, 10.1136/bmjopen-2022-061211. PMC9185559 35676016

[14] J. Wardle and R. Pope , “The Psychological Costs of Screening for Cancer,” Journal of Psychosomatic Research 36, no. 7 (1992): 609–624, 10.1016/0022-3999(92)90051-3. 1403996

[15] A. Miles , J. Wardle , and W. Atkin , “Receiving a Screen‐Detected Diagnosis of Cancer: The Experience of Participants in the UK Flexible Sigmoidoscopy Trial,” Psycho‐Oncology 12, no. 8 (2003): 784–802, 10.1002/pon.705. 14681952

[16] A. R. Cotter , K. Vuong , L. L. Mustelin , et al., “Do Psychological Harms Result From Being Labelled With an Unexpected Diagnosis of Abdominal Aortic Aneurysm or Prostate Cancer Through Screening? A Systematic Review,” BMJ Open 7, no. 12 (2017): e017565, 10.1136/bmjopen-2017-017565. PMC5728272 29237653

[17] D. Moher , A. Liberati , J. Tetzlaff , and D. G. Altman , “Preferred Reporting Items for Systematic Reviews and Meta‐Analyses: The PRISMA Statement,” BMJ 339, no. 1 (2009): b2535, 10.1136/bmj.b2535. 19622551 PMC2714657

[18] J. P. T. Higgins , J. Thomas , J. Chandler , et al., Cochrane Handbook for Systematic Reviews of Interventions. version 6.5 (Cochrane, 2024): (updated August 2024).

[19] Q. N. Hong , S. Fàbregues , G. Bartlett , et al., “The Mixed Methods Appraisal Tool (MMAT) Version 2018 for Information Professionals and Researchers,” Education for Information 34, no. 4 (2018): 285–291, 10.3233/EFI-180221.

[20] Ø Ekeberg , H. Skjauff , and R. Kåresen , “Screening for Breast Cancer Is Associated With a Low Degree of Psychological Distress,” Breast 10, no. 1 (2001): 20–24, 10.1054/brst.2000.0177. 14965553

[21] R. Ellman , B. A. Thomas , and R. Ellman , “Is Psychological Wellbeing Impaired in Long Term Survivors of Breast Cancer?,” Journal of Medical Screening 2, no. 1 (1995): 5–9, 10.1177/096914139500200103. 7497147

[22] J. K. Field , S. W. Duffy , D. R. Baldwin , et al., “The UK Lung Cancer Screening Trial: A Pilot Randomised Controlled Trial of Low‐Dose Computed Tomography Screening for the Early Detection of Lung Cancer,” Health Technology Assessment 20, no. 40 (2016): 1–146, 10.3310/hta20400. PMC4904185 27224642

[23] A. Gibbons , A. Groarke , R. Curtis , and J. Groarke , “The Effect of Mode of Detection of Breast Cancer on Stress and Distress,” Psycho‐Oncology 26, no. 6 (2017): 787–792, 10.1002/pon.4227. 27449013

[24] A. Gibbons , A. Groarke , and K. Sweeney , “Predicting General and Cancer‐Related Distress in Women With Newly Diagnosed Breast Cancer,” BMC Cancer 16, no. 1 (2016): 935, 10.1186/s12885-016-2964-z. 27914469 PMC5135827

[25] C. Lampic , E. Thurfjell , J. Bergh , and P. O. Sjödén , “Short‐ and Long‐Term Anxiety and Depression in Women Recalled After Breast Cancer Screening,” European Journal of Cancer 37, no. 4 (2001): 463–469, 10.1016/S0959-8049(00)00426-3. 11267855

[26] C. Lampic , E. Thurfjell , J. Bergh , M. Carlsson , and P. Sjödén , “Life Values Before Versus After a Breast Cancer Diagnosis,” Research in Nursing & Health 25, no. 2 (2002): 89–98, 10.1002/nur.10029. 11933003

[27] J. L. McSweeney , D. O’Mahony , J. E. Battley , E. Lee , L. Nagle , and S. O’Reilly , “The Impact of Mode of Presentation on Distress in Patients With Early Stage Breast Cancer,” Irish Journal of Medical Science 186, no. 1 (2017): 69–71, 10.1007/s11845-016-1543-2. 28064426

[28] B. I. Schou , R. Kåresen , P. Skaane , K. S. Engelstad , and Ø Ekeberg , “Recall Mammography and Psychological Distress,” European Journal of Cancer 49, no. 4 (2013): 805–811, 10.1016/j.ejca.2012.09.001. 23021930

[29] J. Barrett , V. Jenkins , V. Farewell , et al., “Psychological Morbidity Associated With Ovarian Cancer Screening: Results From More Than 23 000 Women in the Randomised Trial of Ovarian Cancer Screening (UKCTOCS),” BJOG: An International Journal of Obstetrics & Gynaecology 121, no. 9 (2014): 1071–1079, 10.1111/1471-0528.12870. 24865441

[30] I. F. Gareen , F. Duan , E. M. Greco , et al., “Impact of Lung Cancer Screening Results on Participant Health‐Related Quality of Life and State Anxiety in the National Lung Screening Trial,” Cancer 120, no. 21 (2014): 3401–3409, 10.1002/cncr.28833. 25065710 PMC4205265

[31] R. C. N. van den Bergh , I. J. Korfage , M. J. Roobol , et al., “Sexual Function With Localized Prostate Cancer: Active Surveillance Vs Radical Therapy,” BJU International 110, no. 7 (2012): 1032–1039, 10.1111/j.1464-410X.2011.10846.x. 22260273

[32] A. F. W. van der Steeg , C. M. G. Keyzer‐Dekker , J. De Vries , and J. A. Roukema , “Effect of Abnormal Screening Mammogram on Quality of Life,” Journal of British Surgery 98, no. 4 (2011): 537–542, 10.1002/bjs.7371. 21656719

[33] L. D. F. Venderbos , S. Aluwini , M. J. Roobol , et al., “Long‐Term Follow‐Up After Active Surveillance or Curative Treatment: Quality‐of‐Life Outcomes of Men With Low‐Risk Prostate Cancer,” Quality of Life Research 26, no. 6 (2017): 1635–1645, 10.1007/s11136-017-1507-7. 28168601 PMC5420369

[34] F. J. Drummond , E. O’Leary , A. Gavin , H. Kinnear , and L. Sharp , “Mode of Prostate Cancer Detection Is Associated With the Psychological Wellbeing of Survivors: Results From the Picture Study,” Supportive Care in Cancer 24, no. 5 (2016): 2297–2307, 10.1007/s00520-015-3033-x. 26594035 PMC4805717

[35] E. Varela‐Moreno , F. Rivas‐Ruiz , M. Padilla‐Ruiz , et al., “Influence of Depression on Survival of Colorectal Cancer Patients Drawn From a Large Prospective Cohort,” Psycho‐Oncology 31, no. 10 (2022): 1762–1773, 10.1002/pon.6018. 35988209

[36] A. Miles , P. L. McClements , R. J. C. Steele , C. Redeker , N. Sevdalis , and J. Wardle , “The Psychological Impact of a Colorectal Cancer Diagnosis Following a Negative Fecal Occult Blood Test Result,” Cancer Epidemiology Biomarkers & Prevention 24, no. 7 (2015): 1032–1038, 10.1158/1055-9965.EPI-15-0004. 25924826

[37] E. B. Roth , D. B. Jeffe , J. A. Margenthaler , et al., “Method of Breast Cancer Presentation and Depressed Mood 1 Year After Diagnosis in Women With Locally Advanced Disease,” Annals of Surgical Oncology 16, no. 6 (2009): 1637–1641, 10.1245/s10434-009-0445-1. 19360452 PMC3982328

[38] N. Booth , P. Rissanen , T. L. J. Tammela , L. Määttänen , K. Taari , and A. Auvinen , “Health‐Related Quality of Life in the Finnish Trial of Screening for Prostate Cancer,” European Urology 65, no. 1 (2014): 39–47, 10.1016/j.eururo.2012.11.041. 23265387

[39] N. C. A. Vermeer , M. J. M. van der Valk , H. S. Snijders , et al., “Psychological Distress and Quality of Life Following Positive Fecal Occult Blood Testing in Colorectal Cancer Screening,” Psycho‐Oncology 29, no. 6 (2020): 1084–1091, 10.1002/pon.5381. 32237002 PMC7317528

[40] B. Braun , M.‐A. Kurosinski , L. Khil , J. Tio , B. Krause‐Bergmann , and H. W. Hense , “The Mode of Detection is Not Associated With Quality of Life in Women With Breast Cancer,” Breast Care 15, no. 5 (2020): 498–505, 10.1159/000504662. 33223993 PMC7650097

[41] H. Dang , W. H. de Vos tot Nederveen Cappel , S. M. S. van der Zwaan , et al., “Quality of Life and Fear of Cancer Recurrence in T1 Colorectal Cancer Patients Treated With Endoscopic or Surgical Tumor Resection,” Gastrointestinal Endoscopy 89, no. 3 (2019): 533–544, 10.1016/j.gie.2018.09.026. 30273589

[42] R. Ellman , N. Angeli , A. Christians , S. Moss , J. Chamberlain , and P. Maguire , “Psychiatric Morbidity Associated With Screening for Breast Cancer,” British Journal of Cancer 60, no. 5 (1989): 781–784, 10.1038/bjc.1989.359. 2803955 PMC2247323

[43] U. Menon , A. Gentry‐Maharaj , M. Burnell , et al., “Mortality Impact, Risks, and Benefits of General Population Screening for Ovarian Cancer: The UKCTOCS Randomised Controlled Trial,” Health Technology Assessment 29, no. 10 (2023): 1–93, 10.3310/BHBR5832. PMC10542866 37183782

[44] C. C. Burgess , A. J. Ramirez , M. A. Richards , and H. Potts , “Does the Method of Detection of Breast Cancer Affect Subsequent Psychiatric Morbidity?,” European Journal of Cancer 38, no. 12 (2002): 1622–1625, 10.1016/S0959-8049(02)00132-6. 12142052

[45] P. Haddad , P. Maguire , and B. Jones , “Effect of Mode of Breast Cancer Diagnosis on Subsequent Affective Disorder,” Breast 3, no. 4 (1994): 218–221, 10.1016/0960-9776(94)90049-3.

[46] D. Lu , B. Andrae , U. Valdimarsdóttir , et al., “Psychologic Distress Is Associated With Cancer‐Specific Mortality Among Patients With Cervical Cancer,” Cancer Research 79, no. 15 (2019): 3965–3972, 10.1158/0008-5472.CAN-19-0116. 31253667

[47] J. Politi , M. Sala , L. Domingo , et al., “Readmissions and Complications in Breast Ductal Carcinoma in Situ: A Retrospective Study Comparing Screen‐ and Non‐Screen‐Detected Patients,” Women's Health 16 (2020): 1745506520965899, 10.1177/1745506520965899. PMC7594253 33076785

[48] O. Gustafsson , T. Theorell , U. Norming , A. Perski , M. Öhström , and C. Nyman , “Psychological Reactions in Men Screened for Prostate Cancer,” British Journal of Urology 75, no. 5 (1995): 631–636, 10.1111/j.1464-410X.1995.tb07422.x. 7613800

[49] J. Fortin , M. Rivest‐Beauregard , C. Defer , et al., “The Impact of Canadian Medical Delays and Preventive Measures on Breast Cancer Experience: A Silent Battle Masked by the COVID‐19 Pandemic,” Canadian Journal of Nursing Research 55, no. 1 (2023): 55–67, 10.1177/08445621221097520. PMC9086203 35484788

[50] J. H. Yang , V. Huynh , L. D. Leonard , et al., “Are Diagnostic Delays Associated With Distress in Breast Cancer Patients?,” Breast Care 18, no. 4 (2023): 240–248, 10.1159/000529586. 37900555 PMC10601706

[51] M. Solbjør , S. Forsmo , J.‐A. Skolbekken , V. Siersma , and J. Brodersen , “Psychosocial Consequences Among Women With False‐Positive Results After Mammography Screening in Norway,” Scandinavian Journal of Primary Health Care 36, no. 4 (2018): 380–389, 10.1080/02813432.2018.1523985. 30296861 PMC6381538

[52] S. Chinn , “A Simple Method for Converting an Odds Ratio to Effect Size for Use in Meta‐Analysis,” Statistics in Medicine 19, no. 22 (2000): 3127–3131, 10.1002/1097-0258(20001130)19:22<3127::AID-SIM784>3.0.CO;2-M. 11113947

[53] S. Orbell , I. O'Sullivan , R. Parker , B. Steele , C. Campbell , and D. Weller , “Illness Representations and Coping Following an Abnormal Colorectal Cancer Screening Result,” Social Science & Medicine 67, no. 9 (2008): 1465–1474, 10.1016/j.socscimed.2008.06.039. 18687511

[54] G. X. Wu , D. J. Raz , L. Brown , and V. Sun , “Psychological Burden Associated With Lung Cancer Screening: A Systematic Review,” Clinical Lung Cancer 17, no. 5 (2016): 315–324, 10.1016/j.cllc.2016.03.007. 27130469 PMC5606246

[55] I. Henselmans , V. S. Helgeson , H. Seltman , J. de Vries , R. Sanderman , and A. V. Ranchor , “Identification and Prediction of Distress Trajectories in the First Year After a Breast Cancer Diagnosis,” Health Psychology 29, no. 2 (2010): 160–168, 10.1037/a0017806. 20230089

[56] A. W. Boyes , A. Girgis , C. A. D'Este , A. C. Zucca , C. Lecathelinais , and M. L. Carey , “Prevalence and Predictors of the Short‐Term Trajectory of Anxiety and Depression in the First Year After a Cancer Diagnosis: A Population‐Based Longitudinal Study,” Journal of Clinical Oncology 31, no. 21 (2013): 2724–2729, 10.1200/JCO.2012.44.7540. 23775970

[57] S. A. Cook , P. Salmon , G. Hayes , A. Byrne , and P. L. Fisher , “Predictors of Emotional Distress a Year or More After Diagnosis of Cancer: A Systematic Review of the Literature,” Psycho‐Oncology 27, no. 3 (2018): 791–801, 10.1002/pon.4601. 29318702 PMC5873392

[58] A. Irzaldy , J. D. M. Otten , L. M. Kregting , et al., “Quality of Life of Women With a Screen‐Detected Versus Clinically Detected Breast Cancer in the Netherlands: A Prospective Cohort Study,” Quality of Life Research 34, no. 1 (January 2025): 161–171, 10.1007/s11136-024-03783-0. 39287764 PMC11802699

[59] J. Levman and Y. Y. Broza , “Cancer Screening: Recent Developments and Future Directions,” Scientific Reports 15, no. 1 (2025): 9436, 10.1038/s41598-025-89820-5. 40108205 PMC11923198

[60] C. Coens , M. Pe , A. C. Dueck , et al., “International Standards for the Analysis of Quality‐of‐Life and Patient‐Reported Outcome Endpoints in Cancer Randomised Controlled Trials: Recommendations of the SISAQOL Consortium,” Lancet Oncology 21, no. 2 (2020): e83–e96, 10.1016/S1470-2045(19)30790-9. 32007209

