# Supplementary material for: The Psychological Impact of Screen‐Detected Cancer: A Systematic Review
Source: Psychooncology. 2026 Jan 23;35(1):e70358. doi: 10.1002/pon.70358 (PMC12830520; doi:10.1002/pon.70358)
Supplement: Supplementary file 1 — Supporting Information S1 [file PON-35-e70358-s001.docx]

**Appendix 1. Searches**

*Table A1. Embase search strategy*

| # | Search | Results |
| --- | --- | --- |
| 1 | exp anxiety/ | 319134 |
| 2 | exp depression/ | 668429 |
| 3 | exp fear/ | 387530 |
| 4 | (anxiety or depression or worry or distress or psychological impact).tw. | 1010687 |
| 5 | 1 or 2 or 3 or 4 | 1372503 |
| 6 | screen*.tw. | 1465072 |
| 7 | exp cancer screening/ | 104307 |
| 8 | 6 or 7 | 1490077 |
| 9 | diagnos*.tw. | 4701056 |
| 10 | cancer diagnosis/ or tumor diagnosis/ or cancer cytodiagnosis/ or cancer screening/ or cancer test/ or early cancer diagnosis/ | 312164 |
| 11 | 9 or 10 | 4842050 |
| 12 | [positive.tw](https://pubmed.ncbi.nlm.nih.gov/). | 2784268 |
| 13 | 11 or 12 | 6954147 |
| 14 | ("colorectal cancer*" or "breast cancer*" or "prostate cancer*" or "lung cancer*" or "ovarian cancer*" or "cervical cancer*").tw. | 1425138 |
| 15 | exp colorectal cancer/ | 409658 |
| 16 | exp breast cancer/ | 604620 |
| 17 | exp prostate cancer/ | 281906 |
| 18 | exp lung cancer/ | 457591 |
| 19 | exp ovary cancer/ | 159349 |
| 20 | 14 or 15 or 16 or 17 or 18 or 19 | 1988557 |
| 21 | 5 and 8 and 13 and 20 | 5181 |

*Table A2. PsycINFO search strategy*

| # | Search | Results |
| --- | --- | --- |
| 1 | exp anxiety/ | 97050 |
| 2 | exp depression (emotion)/ | 27266 |
| 3 | exp fear/ | 21777 |
| 4 | (anxiety or depression or worry or distress or psychological impact).tw. | 532375 |
| 5 | 1 or 2 or 3 or 4 | 555229 |
| 6 | screen*.tw. | 129426 |
| 7 | exp cancer screening/ | 5912 |
| 8 | 6 or 7 | 130127 |
| 9 | diagnos*.tw. | 383422 |
| 10 | diagnosis/ or screening/ | 64635 |
| 11 | 9 or 10 | 394233 |
| 12 | [positive.tw](http://positive.tw/). | 467379 |
| 13 | 11 or 12 | 827242 |
| 14 | ("colorectal cancer*" or "breast cancer*" or "prostate cancer*" or "lung cancer*" or "ovarian cancer*" or "cervical cancer*").tw. | 27179 |
| 15 | exp Breast Neoplasms/ | 12138 |
| 16 | 14 or 15 | 27507 |
| 17 | 5 and 8 and 13 and 16 | 509 |

*Table A3. PubMed/MEDLINE search strategy*

| # | Search | Results |
| --- | --- | --- |
| 1 | anxiety[MeSH Terms] | 119,417 |
| 2 | depression[MeSH Terms] | 267,925 |
| 3 | fear[MeSH Terms] | 41,613 |
| 4 | anxiety[Title/Abstract] OR depression[Title/Abstract] OR worry[Title/Abstract] OR distress[Title/Abstract] OR "psychological impact"[Title/Abstract] | 751,918 |
| 5 | #1 OR #2 OR #3 OR #4 | 861,603 |
| 6 | screen*[Title/Abstract] | 1,055,025 |
| 7 | cancer screening[MeSH Terms] | 41,034 |
| 8 | #6 or #7 | 1,065,679 |
| 9 | diagnos*[Title/Abstract] | 3,274,556 |
| 10 | [positive.tw](http://positive.tw/).  UPDATED SEARCH: **positive[tw]** | 2,115,704 |
| 11 | #9 OR #10 | 5,001,288 |
| 12 | "Colorectal cancer*"[Title/Abstract] OR "Breast cancer*"[Title/Abstract] OR "prostate cancer*"[Title/Abstract] OR "lung cancer*"[Title/Abstract] OR "ovarian cancer*"[Title/Abstract] OR "cervical cancer*"[Title/Abstract] | 976,770 |
| 13 | breast neoplasm[MeSH Terms] | 356,366 |
| 14 | cancer, colorectal[MeSH Terms] | 247,785 |
| 15 | lung neoplasm[MeSH Terms] | 286,871 |
| 16 | prostate neoplasm[MeSH Terms] | 154,387 |
| 17 | cervical neoplasm[MeSH Terms] | 88,187 |
| 18 | ovarian neoplasm[MeSH Terms] | 98,728 |
| 19 | #12 OR #13 OR #14 OR #15 OR #16 OR #17 | 1,408,032 |
| 20 | #18 OR #19 | 1,447,487 |
| 21 | #5 AND #8 AND #11 AND #20 | [1,530](https://pubmed.ncbi.nlm.nih.gov/?term=%235+AND+%238+AND+%2311+AND+%2320&sort=) |

**Appendix 2. Data extraction form content**


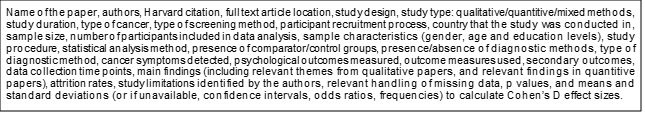


**Appendix 3. Full results of quality appraisal using the Mixed Methods Appraisal Tool**

| **Study** | **Rating** | **1.1** | **1.2** | **1.3** | **1.4** | **1.5** | **2.1** | **2.2** | **2.3** | **2.4** | **2.5** | **3.1** | **3.2** | **3.3** | **3.4** | **3.5** | **4.1** | **4.2** | **4.3** | **4.4** | **4.5** | **5.1** | **5.2** | **5.3** | **5.4** | **5.5** |
| --- | --- | --- | --- | --- | --- | --- | --- | --- | --- | --- | --- | --- | --- | --- | --- | --- | --- | --- | --- | --- | --- | --- | --- | --- | --- | --- |
| Barrett et al. | 80% (high) |  |  |  | 1 | 1 | 1 | 0 | 1 |  |  |  |  |  |  |  |  |  |  | 1 | 1 | 1 | 0 | 1 |  |  |
| Booth et al. | 80% (high) |  |  |  | 1 | 1 | 0 | 1 | 1 |  |  |  |  |  |  |  |  |  |  | 1 | 1 | 0 | 1 | 1 |  |  |
| Braun et al. | 100% (high) |  |  |  |  |  | 1 | 1 | 1 | 1 | 1 |  |  |  |  |  |  |  |  |  |  | 1 | 1 | 1 | 1 | 1 |
| Burgess et al. | 80% (high) |  |  |  |  |  | 0 | 1 | 1 | 1 | 1 |  |  |  |  |  |  |  |  |  |  | 0 | 1 | 1 | 1 | 1 |
| Dang et al. | 80% (high) |  |  |  |  |  | 0 | 1 | 1 | 1 | 1 |  |  |  |  |  |  |  |  |  |  | 0 | 1 | 1 | 1 | 1 |
| Drummond et al. | 80% (high) |  |  |  |  |  | 0 | 1 | 1 | 1 | 1 |  |  |  |  |  |  |  |  |  |  | 0 | 1 | 1 | 1 | 1 |
| Ekeberg et al. | 60% (medium) |  |  |  |  |  | 1 | 1 | 0 | 0 | 1 |  |  |  |  |  |  |  |  |  |  | 1 | 1 | 0 | 0 | 1 |
| Ellman et al. | 80% (high) |  |  |  |  |  | 1 | 1 | 1 | 0 | 1 |  |  |  |  |  |  |  |  |  |  | 1 | 1 | 1 | 0 | 1 |
| Ellman et al. | 80% (high) |  |  |  |  |  | 0 | 1 | 1 | 1 | 1 |  |  |  |  |  |  |  |  |  |  | 0 | 1 | 1 | 1 | 1 |
| Field et al. | 80% (high) |  |  |  | 1 | 1 | 1 | 0 | 1 |  |  |  |  |  |  |  |  |  |  | 1 | 1 | 1 | 0 | 1 |  |  |
| Fortin et al. | 40% (medium) |  |  |  |  |  |  |  |  |  |  | 1 | 0 | 0 | 0 | 1 |  |  |  |  |  |  |  |  |  |  |
| Gareen et al. | 80% (high) |  |  |  |  |  | 1 | 1 | 1 | 1 | 0 |  |  |  |  |  |  |  |  |  |  | 1 | 1 | 1 | 1 | 0 |
| Gibbons et al. | 40% (medium) |  |  |  |  |  | 0 | 1 | 0 | 0 | 1 |  |  |  |  |  |  |  |  |  |  | 0 | 1 | 0 | 0 | 1 |
| Gibbons et al. | 60% (medium) |  |  |  |  |  |  |  |  | 1 | 0 | 1 | 0 | 1 |  |  |  |  |  |  |  |  |  |  | 1 | 0 |
| Gustafsson et al., (1995), Sweden | 40% (medium) |  |  |  |  |  | 1 | 0 | 0 | 0 | 1 |  |  |  |  |  |  |  |  |  |  | 1 | 0 | 0 | 0 | 1 |
| Haddad et al. | 100% (high) |  |  |  |  |  | 1 | 1 | 1 | 1 | 1 |  |  |  |  |  |  |  |  |  |  | 1 | 1 | 1 | 1 | 1 |
| Lampic et al. | 80% (high) |  |  |  |  |  | 0 | 1 | 1 | 1 | 1 |  |  |  |  |  |  |  |  |  |  | 0 | 1 | 1 | 1 | 1 |
| Lampic et al. | 60% (medium) |  |  |  |  |  | 0 | 1 | 1 | 0 | 1 |  |  |  |  |  |  |  |  |  |  | 0 | 1 | 1 | 0 | 1 |
| Lu et al. | 100% (high) |  |  |  |  |  | 1 | 1 | 1 | 1 | 1 |  |  |  |  |  |  |  |  |  |  | 1 | 1 | 1 | 1 | 1 |
| McSweeney et al., (2017), Ireland | 80% (high) |  |  |  |  |  | 0 | 1 | 1 | 1 | 1 |  |  |  |  |  |  |  |  |  |  | 0 | 1 | 1 | 1 | 1 |
| Menon et al. | 80% (high) |  |  |  | 1 | 1 | 1 | 0 | 1 |  |  |  |  |  |  |  |  |  |  | 1 | 1 | 1 | 0 | 1 |  |  |
| Miles et al. | 80% (high) |  |  |  |  |  | 1 | 1 | 0 | 1 | 1 |  |  |  |  |  |  |  |  |  |  | 1 | 1 | 0 | 1 | 1 |
| Orbell et al. | 60% (medium) |  |  |  |  |  | 0 | 1 | 0 | 1 | 1 |  |  |  |  |  |  |  |  |  |  | 0 | 1 | 0 | 1 | 1 |
| Politi et al. | 80% (high) |  |  |  |  |  | 0 | 1 | 1 | 1 | 1 |  |  |  |  |  |  |  |  |  |  | 0 | 1 | 1 | 1 | 1 |
| Roth et al. | 80% (high) |  |  |  |  |  | 1 | 1 | 0 | 1 | 1 |  |  |  |  |  |  |  |  |  |  | 1 | 1 | 0 | 1 | 1 |
| Solbjør et al. | 80% (high) |  |  |  |  |  | 1 | 1 | 0 | 1 | 1 |  |  |  |  |  |  |  |  |  |  | 1 | 1 | 0 | 1 | 1 |
| Schou Bredal et al. | 80% (high) |  |  |  |  |  | 0 | 1 | 1 | 1 | 1 |  |  |  |  |  |  |  |  |  |  | 0 | 1 | 1 | 1 | 1 |
| Van den Bergh et al. | 80% (high) |  |  |  |  |  | 1 | 1 | 0 | 1 | 1 |  |  |  |  |  |  |  |  |  |  | 1 | 1 | 0 | 1 | 1 |
| Van der Steeg et al. | 80% (high) |  |  |  |  |  | 1 | 1 | 0 | 1 | 1 |  |  |  |  |  |  |  |  |  |  | 1 | 1 | 0 | 1 | 1 |
| Varela‐Moreno et al., (2022), Spain | 100% (high) |  |  |  |  |  |  |  |  | 1 | 1 | 1 | 1 | 1 |  |  |  |  |  |  |  |  |  |  | 1 | 1 |
| Venderbos et al. | 80% (medium) |  |  |  |  |  | 1 | 1 | 0 | 1 | 1 |  |  |  |  |  |  |  |  |  |  | 1 | 1 | 0 | 1 | 1 |
| Vermeer et al. | 60% (medium) |  |  |  |  |  |  |  |  | 1 | 0 | 1 | 0 | 1 |  |  |  |  |  |  |  |  |  |  | 1 | 0 |
| Yang et al. | 60% (medium) |  |  |  |  |  | 0 | 1 | 1 | 0 | 1 |  |  |  |  |  |  |  |  |  |  | 0 | 1 | 1 | 0 | 1 |

**Appendix 4. Full study results including data presented by treatment group**

| **First author (year)**  **Cancer type** | **Outcome measure** | **Time points** | **Results for screen detected cancer group as reported** | | |
| --- | --- | --- | --- | --- | --- |
| **ANXIETY** | | | | | |
| **HADS-A** | | | | | |
| Ekeberg (2001)^+^, Breast ^21^ | HADS-A: Seven items  scored 0–3, range 0-21.  >11 indicates anxiety case. | 1. At recall  2. 4 weeks post-diagnosis | **Dx after 3 tests:**  1. M=9.5 (n=13, SD=3.9)  2. M=6.8 (n=13, SD=4.1)  **Dx after biopsy:**  1. M=8.1 (n=12, SD=5.2)  2. M=8.8 (n=12, SD=5.6) | | **Total SnD cancer group:**  1. %>11=28%  2. %>11 = 24% |
| **STAI** | | | | | |
| Gareen (2014)^+^, Lung ^30^ | STAI-20 (state scale): Total score 20-80. Median norm for 50-69 year old men is 34.51, women 32.20 | 1. 1-month post-screening  2. 6-months post-screening  **20/63 diagnosed prior to 1-month time point,*  *21/63 diagnosed prior to 6-month time point.* | **CT arm (n=41)**  1. M=41.06 (SD=15.10)  2. M=37.69 (SD=12.04)  **X-ray arm (n=22)**  1. M=39.43 (SD=11.66)  2. M=39.38 (SD=14.47) | | |
| Van den Bergh (2012)^+^,  Prostate^32^ | STAI-6 (Score range 20 – 80 (maximum  anxiety), threshold for high anxiety: > 44) | 1. 6-months post-diagnosis  2. 18-months post-diagnosis | **Active surveillance (AS)**  1. M = 35.8 (n=129)  2. M = 34.8 (n=78)  **Radiotherapy (RT)**  1. M = 30.3 (n=70)  2. M = 32.0 (n=70)  **Radical prostatectomy (RP)**  1. M = 33.7 (n=67)  2. M = 32.8 (n=67) | | |
| **DEPRESSION** | | | | | |
| **HADS-D** | | | | | |
| Ekeberg (2001)^+,^ Breast ^21^ | HADS-D: Seven items  scored 0–3. Score  calculated by simple addition. >11 indicates ‘caseness’. | 1. At recall  2. 4 weeks post-diagnosis | **Dx after 3 tests:**  1. M=4.3 (n=14, SD=3.4)  2. M=3.8 (n=13, SD=3.6)  **Dx after biopsy:**  1. M=3.7 (n=12, SD=3.4)  2. M=4.9 (n=12, SD=5.2) | | **Total SnD cancer group:**  1. N>11=1 (3.8%)  2. N>11=3 (12%) |
| **CES-D** | | | | | |
| Van den Bergh (2012)^+^,  Prostate^32^ | CES-D (§ Score range 0 – 60 (maximum depression), threshold for clinically depressive: ≥ 16) | 1. 6-months post-diagnosis  2. 18-months post-diagnosis | **Active surveillance (AS)**  1. M = 5.8 (n=129)  2. M = 5.4 (n=78)  **Radiotherapy (RT)**  1. M = 8.6 (n=70)  2. M = 8.4 (n=70)  **Radical prostatectomy (RP)**  1. M = 7.5 (n=67)  2. M = 7.3 (n=67) | | |
| **CANCER-SPECIFIC PSYCHOLOGICAL DISTRESS** | | | | | |
| **Cancer Worry Scale** | | | | | |
| Dang (2019)^+^, Colorectal ^42^ | Cancer Worry Scale: 4 items on 10-point scales, summed for the total score (0-40). Higher scores= more fear of cancer recurrence. | 1. Endoscopy: median 18 months  2. Surgery: median 21 months | 1. Endoscopy: M= 7.6 (SD= 4.5) (N=55)  2. Surgery: M= 9.7 (SD= 8.2) (N=55) | | |
| **QUALITY OF LIFE AND HEALTH STATUS** | | | | | |
| **Short Form-36 or 12** | | | | | |
| Gareen (2014)^+^, Lung ^30^ | SF-36 Physical & Mental Component Scores (PCS & MCS; Score range 0 – 100 (best health), mean score 50, standard deviation 10; Adults aged 55-64, the  median norm PCS is 50.65 & MCS is 55.28. Adults  65-74, the median norm PCS is 46.11 & MCS is  56.11; Clinically important change over time is 3-5 points) | 1. Baseline (first screening appointment)  2. 1-month post-screening  3. 6-months post-screening  **20/63 diagnosed prior to 1-month time point,*  *21/63 diagnosed prior to 6-month time point.* | **CT arm – PCS (n=41)**  1. M=46.59 (SD=11.33)  2. M=44.50 (SD=11.57)  3. M=32.38 (SD=12.57)  **CT arm – MCS (n=41)**  1. M=52.03 (SD=11.04)  2. M=44.14 (SD=14.02)  3. M=46.30 (SD=13.65)  **X-Ray – PCS (n=22)**  1. M=48.52 (SD=9.88)  2. M=42.08 (SD=11.06)  3. M=38.48 (SD=10.04)  **X-Ray – MCS (n=22)**  1. M=53.77 (SD=8.57)  2. M=51.42 (SD=9.89)  3. M=46.22 (SD=12.17) | | |
| Van den Bergh (2012)^+^,  Prostate ^32^ | SF-12 Physical & Mental Component Scores (PCS & MCS; Score range 0 – 100 (best health), mean score is 50 in general  population.) | 1. 6 months post-diagnosis  2. 12-18 months post-diagnosis | **Active surveillance (AS) - PCS**  1. M=50.3 (n=129)  2. M=51.2 (n=78)  **Active surveillance (AS) - MCS**  1. M=54.2 (n=129)  2. M=53.9 (n=78)  **Radiotherapy (RT) - PCS**  1. M=48.0 (n=70)  2. M=47.3 (n=70) | | **Radiotherapy (RT) - MCS**  1. M=52.5 (n=70)  2. M=54.8 (n=70)  **Radical prostatectomy (RP) - PCS**  1. M=51.9 (n=67)  2. M=51.2 (n=67)  **Radical prostatectomy (RP) - MCS**  1. M=53.8 (n=67)  2. M=55.3 (n=67) |
| **EQ-5D** | | | | | |
| Dang (2019)^+^,  Colorectal^42^ | EQ-5D-5L index value (score 0-1) and visual analogue scale (score 0=worst health, 100=best health) | 1. Endoscopy: median 18 months  2. Surgery: median 21 months | 1. VAS M=84.1 (SD=11.7)  Descriptive system M=0.88 (SD=0.11) (n=110)  2. VAS M=85.2 (SD=12.5)  Descriptive system M=0.90 (SD=0.09) (n=55) | | |
| **EORTC QLQ-C30** | | | | | |
| Dang (2019)^+^,  Colorectal^42^ | EORTC QLQ-C30 version 3 (scales scored 0-100, higher scores indicate better outcomes) | 1. Endoscopy: median 18 months  2. Surgery: median 21 months | **Endoscopy group (n=55)**  **Global health:** M=84.4 (SD=14.2)  **Physical** **functioning**: M=92.0 (SD=11.3)  **Role functioning**: M=89.7 (SD=20.7)  **Emotional** **functioning**: M=90.8 (SD=15.2)  **Cognitive** **functioning**: M=91.8 (SD=16.0)  **Social** **functioning**: M=94.8 (SD=13.9)  **Fatigue**: M=11.3 (SD=17.7)  **Nausea** **&** **vomiting**: M=3.3 (SD=10.8)  **Pain**: M=10.0 (SD=16.0)  **Dyspnea**: M=8.5 (SD=17.7)  **Insomnia**: M=13.3 (SD=19.9)  **Appetite** **loss**: M=4.8 (SD=16.3)  **Constipation**: M=5.5 (SD=14.0)  **Diarrhea**: M=7.3 (SD=18.9)  **Financial** **difficulties**: M=2.4 (SD=18.9) | **Surgery group (n=55):**  **Global** **health**: M=83.8 (SD=14.6)  **Physical** **functioning**: M=91.6 (SD=12.3)  **Role** **functioning**: M=92.4 (SD=15.3)  **Emotional** **functioning**: M=87.7 (SD=14.2)  **Cognitive** **functioning**: M=94.5 (SD=11.6)  **Social** **functioning**: M=92.7 (SD=16.3)  **Fatigue**: M=14.3 (SD=18.4)  **Nausea** **&** **vomiting**: M=3.6 (SD=11.0)  **Pain**: M=9.7 (SD=18.6)  **Dyspnea**: M=10.3 (SD=19.1)  **Insomnia**: M=14.5 (SD=22.9)  **Appetite** **loss**: M=3.6 (SD=12.3)  **Constipation**: M=9.7 (SD=21.9)  **Diarrhea**: M=5.5 (SD=14.0)  **Financial** **difficulties**: M=3.0 (SD=11.6) | |

**Appendix 5. Figures**

**
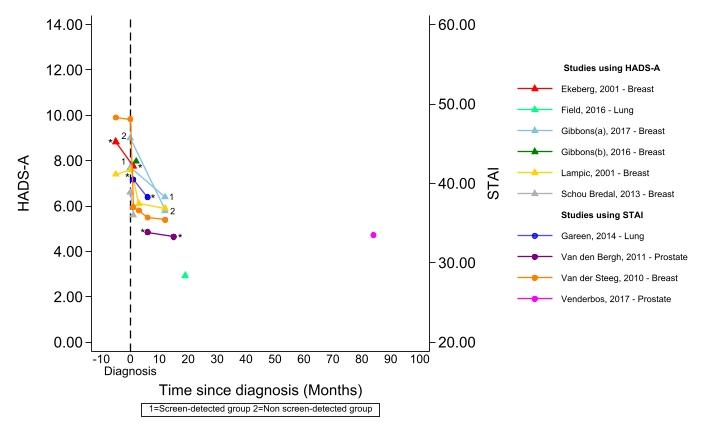
Figure A1. Anxiety among participants with screen-detected and non-screen-detected cancer reported over time using the STAI or HADS-A, including those with more than 24 months follow-up.**

**Anxiety scores were reported for sub-groups of those with screen-detected cancer (e.g. by treatment) so an overall mean has been calculated. Note: Where cut-offs for anxiety were reported, these ranged from >8 to >11 (for HADS-A) and were >44 (for the STAI)*

**
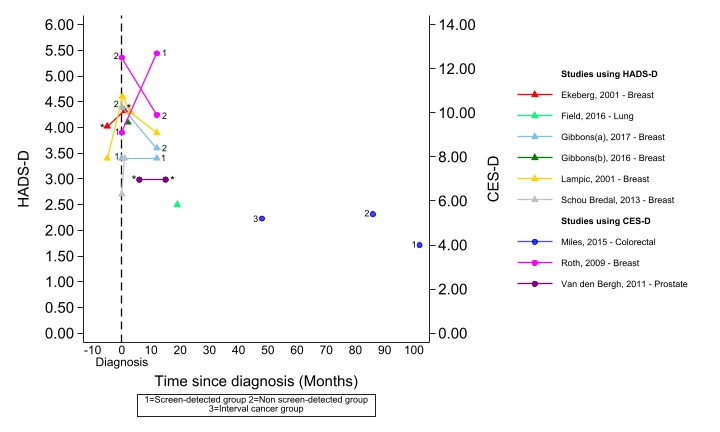
Figure A2. Depression among participants with screen-detected and non-screen-detected cancer reported over time using the HADS-D or CES-D, including those with more than 24 months follow-up.**

**Depression scores were reported for sub-groups of those with screen-detected cancer (e.g. by treatment) so an overall mean was calculated for presentation. Note: Where cut-offs for depression were reported, these ranged from >8 to >14 (for HADS-D) and >16 (for CES-D).*

**Appendix 6. Study results by for other psychological outcomes.**

| **Author (year)**  **cancer type** | **Outcome measure** | **Time points (n)** | **Results for baseline (where applicable) and screen detected cancer group** | **Results for non-screen detected group(s)** | **Difference by mode of detection, [Effect size (Cohen’s D)]** |
| --- | --- | --- | --- | --- | --- |
| **Revised Illness Perception Questionnaire (IPQ-R)** | | | | | |
| Gibbons (2016)  Breast ^24^ | IPQ-R: Items rated 1-5. from 1 (*strongly disagree*) to 5 (*strongly agree*) Higher scores= worse outcomes. Scales have the following number of items: chronic timeline (6), consequences (6), illness coherence (5), identity (pick from list of 19 symptoms), causes (6). | After dx/before tx (n=94) | **Identity:** M=2.25 (SD=2.29) **Chronic timeline:** M=12.74 (SD=4.48) **Consequences:** M=19.67 (SD=4.81) **Personal control:** M=21.96 (SD=4.39) **Illness coherence:** M=17.53 (SD=4.42) **Emotional causes:** M=14.36 (SD=4.65) |  |  |
| Orbell (2008)  Colorectal ^31^ | IPQ-R – adapted version. All items scored on a 6 point scale (disagree very strongly to agree very strongly) except identity bowel symptom score which ranged from 0 to 10. Mean scores were computed for each scale. Higher scores indicated stronger beliefs in a given domain. | 8 months after screening (n=196) | **Identity:** Mean=2.58  **Behavioural attribution:** Mean=2.66  **Biologic attribution:** Mean=3.22  **Psychological attribution:** Mean=2.75  **Timeline:** Mean = 3.35  **Consequences:** Mean=3.35  **Emotional representation:** Mean=3.46  **Illness coherence:** Mean=3.16  **Treatment control:** Mean=4.45  **Personal control:** Mean=3.81 |  |  |
| **Coping** | | | | | |
| Gibbons (2016)  Breast ^24^ | Mental Adjustment to Cancer (MAC) Fighting Spirit (17 items) and Anxious Preoccupation (9 items) subscales. Scored 1-4. Higher scores = better coping. | After dx/before tx (n=94) | **Fighting spirit:** Mean=46 (SD=5.28) **Anxious preoccupation:** Mean=21.96 (SD=3.86) | - | - |
| Orbell (2008)  Colorectal ^31^ | Ways of coping questionnaire- 4-point scale (not used to used a great deal). Higher scores = change towards healthier behaviour. | 8 months after screening (n=196) | Not presented | - | - |
| **Decisional regret** | | | | | |
| Field (2016)  Lung ^23^ | Satisfaction with Decision Scale: 6 items scored 1-5. Average is total score (1-5). Scores <5= ‘not very satisfied’, scores=5 ‘very satisfied’. | 1. Baseline before CT scan (people later invited to screening n=2018)  2. Two weeks after initial scan (positive referrals, n=42)  3. 10-27 months later (true positives, n=21) | 1. N<5=1228 (61%)  2. N<5=18 (43%)  3. N<5=6 (29%) | - | - |
| Vermeer (2020)  Colorectal ^41^ | Decisional regret scale: 5 items with likert-scale responses. Total score 0-100. Scores >25 = high regret. | 1. Post-colonoscopy (n=60)  2. 6 months (n=49) | Not reported for SD cancer alone at specific time points.  %>25=10% | - | - |
| **Perceived risk of recurrence** | | | | | |
| Roth (2009)  Breast ^39^ | Perceived risk - categories: 0%, 1-9%, 10-24%, 25-49%, 50% and >50%. Interpretation not described.  (At both timeloints- SnD: n=86; non-SnD: n=75) | 1. At diagnosis  2. 1-year post-diagnosis | 1. Not measured  2. 0% chance recurrence=9.1%  1-9%= 18.2%; 10-24%= 9.1%; 25-49%= 18.2%; 50%= 36.4%; >50%= 9.1% | 1. Not measured  2. 0% chance recurrence=19.5%;  1-9%= 9.1%; 10-24%= 14.3%; 25-49%= 18.2%; 50%= 19.5%; >50%= 16.9% | No significant difference  p=0.68  [SD vs symptomatic: 0.13 |
| **Live values – Bespoke life values questionnaire** | | | | | |
| Lampic (2002)  Breast ^27^ | Life values questionnaire – 7 life value factors (present and future)- Likert scale 1 to 7 - Mean life value indexes computed. Negative scores indicate that the perceived importance of a specific value is higher than the attainment of that value. | 1. 3 months after recall (n=34)  2. 1 year after recall (n=31) | Descriptive results not presented | - | - |
| **Personality - NEO-FFI** | | | | | |
| van der Steeg (2011)  Breast ^33^ | NEO-FFI – scoring and interpretation not described | 1. Before diagnosis  2. At diagnosis  3. 1-month post-diagnosis  4. 3-months post-diagnosis  5. 6-months post-diagnosis  6. 12-months post-diagnosis  (n=152, not reported per timepoint) | **1. Neuroticism:** Mean=30.0 (SD=7.2)  **Extraversion**: Mean=40.8 (SD=5.4)  **Openness**: Mean=35.6 (SD=6.0)  **Agreeableness**: Mean=43.9 (SD=4.1)  **Conscientiousness**: Mean=45.7 (SD=5.4)  **Trait anxiety:** Mean=39.6 (SD=11.2)  *Not collected at other time points |  |  |
| **Perceived diagnostic delay** | | | | | |
| Miles (2015), Colorectal ^38^ | Assessed with the item: “Do you think your cancer could have been diagnosed sooner than it was” with response options: “yes,” “no,” and “not sure.” | 5-12 years post-diagnosis  (SnD: n=106; interval: n=99; no screening: n=91) | N yes=15 (14%) | **Interval:**  N yes=30 (31%)  **No screening:**  N yes=34 (37%) | Interval perceived significantly more delay than SD (adjusted); Difference=-0.37 (CI=0.17-0.83; p=0.02)  [SD vs interval: -0.54  SD vs no screening: -0.58] |
| **Trust in FOBt screening results** | | | | | |
| Miles (2015), Colorectal ^38^ | Assessed with the item: “If you were to have an FOB test, would you trust the results of the test” with response options: “not at all,” “somewhat,” “moderately,” and “very much” – 4 point scale, scored from 1-4 | 5-12 years post-diagnosis  (SnD: n=105; interval: n=99; no screening: n=89) | Mean= 3.84 (n=105, SD=0.46) | Interval:  Mean=2.74 (n=99, SD=1.06)  No screening:  Mean=3.60 (n=89, SD=0.56) | Interval had significantly less trust than SD (adjusted); Difference=-1.11 (CI=0.87-1.35; p<0.001)  Interval had significantly less trust than no screening (adjusted)  Difference=0.86 (CI=0.63-1.09; p<0.001)  [SD vs interval: 1.36  SD vs no screening: 0.47] |
| **Sleep Disturbance Index (SDI)** | | | | | |
| Gustafsson (1995)  Prostate ^50^ | SDI: 10 items (scored 1-4), mean of summed scores calculated. | 1. Screening (all) (n=100)  2. Biopsy 2 weeks after screening (includes all participants) (n=307)  3. 4 weeks after screening (cancer only) (n=34)  4. 16 weeks after screening (cancer only) (n=33) | 1. Mean=1.90 (CI=1.79-2.01; n=99)  2. Mean=1.94 (CI=1.67-2.21; n=307)  3. Mean=2.14 (CI=1.86-2.42; n=34)  4. Mean=1.94 (CI=1.80-2.18; n=33) | - | - |
